# A region of suppressed recombination misleads neoavian phylogenomics

**DOI:** 10.1073/pnas.2319506121

**Published:** 2024-04-01

**Authors:** Siavash Mirarab, Iker Rivas-González, Shaohong Feng, Josefin Stiller, Qi Fang, Uyen Mai, Glenn Hickey, Guangji Chen, Nadolina Brajuka, Olivier Fedrigo, Giulio Formenti, Jochen B. W. Wolf, Kerstin Howe, Agostinho Antunes, Mikkel H. Schierup, Benedict Paten, Erich D. Jarvis, Guojie Zhang, Edward L. Braun

**Affiliations:** ^a^Electrical and Computer Engineering Department, University of California, San Diego, CA 95032; ^b^Bioinformatics Research Centre, Aarhus University, Aarhus 8000, Denmark; ^c^Center for Evolutionary & Organismal Biology, Zhejiang University School of Medicine, Hangzhou 310058, China; ^d^Liangzhu Laboratory, Zhejiang University, Hangzhou 311121, China; ^e^Section for Ecology & Evolution, Department of Biology, University of Copenhagen, København 2100, Denmark; ^f^BGI-Research, Shenzhen 518083, China; ^g^Genomics Institute, University of California, Santa Cruz, CA 96064; ^h^Vertebrate Genome Lab, Rockefeller University, New York, NY 10065; ^i^Division of Evolutionary Biology, Faculty of Biology, Ludwig-Maximillians-Universität, Munich 82152, Germany; ^j^Tree of Life Division, Wellcome Sanger Institute, Cambridge CB10 1RQ, United Kingdom; ^k^Interdisciplinary Centre of Marine and Environmental Research, University of Porto, Porto 4099-002, Portugal; ^l^Department of Biology, Faculty of Sciences, University of Porto, Porto 4099-002, Portugal; ^m^Department of Biology, University of Florida, Gainesville, FL 32611

**Keywords:** phylogenetic discordance, recombination, avian phylogeny, genome rearrangement, phylogenomics

## Abstract

Genomes are typically mosaics of regions with different evolutionary histories. When speciation events are closely spaced in time, recombination makes the regions sharing the same history small, and the evolutionary history changes rapidly as we move along the genome. When examining rapid radiations such as the early diversification of Neoaves 66 Mya, typically no consistent history is observed across segments exceeding kilobases of the genome. Here, we report an exception. We found that a 21-Mb region in avian genomes, mapped to chicken chromosome 4, shows an extremely strong and discordance-free signal for a history different from that of the inferred species tree. Such a strong discordance-free signal, indicative of suppressed recombination across many millions of base pairs, is not observed elsewhere in the genome for any deep avian relationships. Although long regions with suppressed recombination have been documented in recently diverged species, our results pertain to relationships dating circa 65 Mya. We provide evidence that this strong signal may be due to an ancient rearrangement that blocked recombination and remained polymorphic for several million years prior to fixation. We show that the presence of this region has misled previous phylogenomic efforts with lower taxon sampling, showing the interplay between taxon and locus sampling. We predict that similar ancient rearrangements may confound phylogenetic analyses in other clades, pointing to a need for new analytical models that incorporate the possibility of such events.

## Significance

Genomes are mosaics of evolutionary histories, and over time, regions of shared history shrink due to recombination. We typically observe frequent changes in evolutionary trees across the genome, especially for rapid radiations. We have found an exception across 21 Mb of neoavian genomes. Unexpectedly, this region shows a consistent history for the first divergence among Neoaves circa 65 Mya. Moreover, the history strongly supported in this region differs from the inferred species tree. We show that the cause of this surprising pattern may be an ancient rearrangement that remained polymorphic across multiple speciation events. We demonstrate that this single region can interact with limited taxon sampling to mislead phylogenomic analyses.

The potential for conflicting evolutionary histories across the genome, often called gene tree–species tree discordance (1), has now been fully incorporated into evolutionary theory (2). This change reflects the plethora of genome-wide analyses that have documented discordance across the genome, starting from early such analyses (3). Besides inference error (4–6), there are several causes for true biological discordance. Incomplete lineage sorting (ILS) is an omnipresent source of discordance (7–10), and it can be exacerbated by hybridization (11). ILS is a by-product of neutral evolution and the presence of polymorphisms in populations that undergo successive speciations. The random sorting of polymorphisms into descendent lineages may not match the species tree (12). Thus, ILS, which occurs with a nonzero probability for every recombining genome, has been the default biological explanation for observed discordance and has been targeted by many methods of species tree inference (13). Discordance due to hybridization does not impact all branches of the tree but can be very common in some clades (14, 15) and is observed in birds (16); however, hybridization is not strictly an alternative to ILS as deep coalescences can occur on phylogenetic networks just as they do on trees.

An important signature of ILS is its randomness. Evolutionary trees for individual loci represent different realizations of a stochastic process, captured by the multi-species coalescence (MSC) model (17). ILS is expected to be present across the genome, and contiguous windows with the same history are expected to be short due to accumulated recombinations, reaching an expected equilibrium of $1/\left(2N_{e}r\right)$ base pairs (bp). While estimates of recombination rate r and effective population size $N_{e}$ vary, using reasonable ranges for birds (e.g., $10^{5}\leq N_{e}\leq 10^{6}$ and $1.2\times 10^{-9}\leq r\leq 10^{-7}$ per bp; see refs. 18 and 19), these windows can range between 5 bp and 4,000 bp. Thus, at the higher end, the recombination-free window sizes measure in thousands of base pairs and not millions. As a consequence, for sufficiently short branches of the species tree (which have experienced high levels of ILS), we expect the evolutionary history to change frequently as we move along the genome; for such branches, it would be exceedingly unlikely that long stretches of the genome (e.g., > 1 Mbp) would have evolved under the same topology, displaying no discordance. Note that genomic segments with different histories do not necessarily follow the boundaries between genes, and hence, we will use the term “locus trees,” as opposed to the typically used gene tree.

The early radiation of Neoaves, the clade comprising ca. 95% of bird species (20), has extensive phylogenetic discordance, often attributed to abundant ILS (10, 21). Quantifying levels of ILS has been difficult due to the confounding effect of stochastic error and systematic bias in locus tree estimation (4–6). Nevertheless, the signatures of ILS among early divergences of Neoaves are observed regardless of the data type (e.g., both coding and noncoding sequences) used for phylogenetic estimation. Analyses of other genomic changes, such as insertions and deletions, also provide strong evidence for ILS in the early branches of Neoaves (10, 22, 23). Although rare genomic changes can exhibit homoplasy (see ref. 24.for a transposable element example), most conflicts between these low homoplasy characters and the species tree are likely to reflect ILS. This combination of challenges has motivated genome-wide studies of bird evolution (25–27), including whole-genome analyses by Jarvis et al. (10), which included 48 species representing most bird orders, and a recent study by Stiller et al. (21), which included 363 species representing most bird families.

**Fig. 1.**
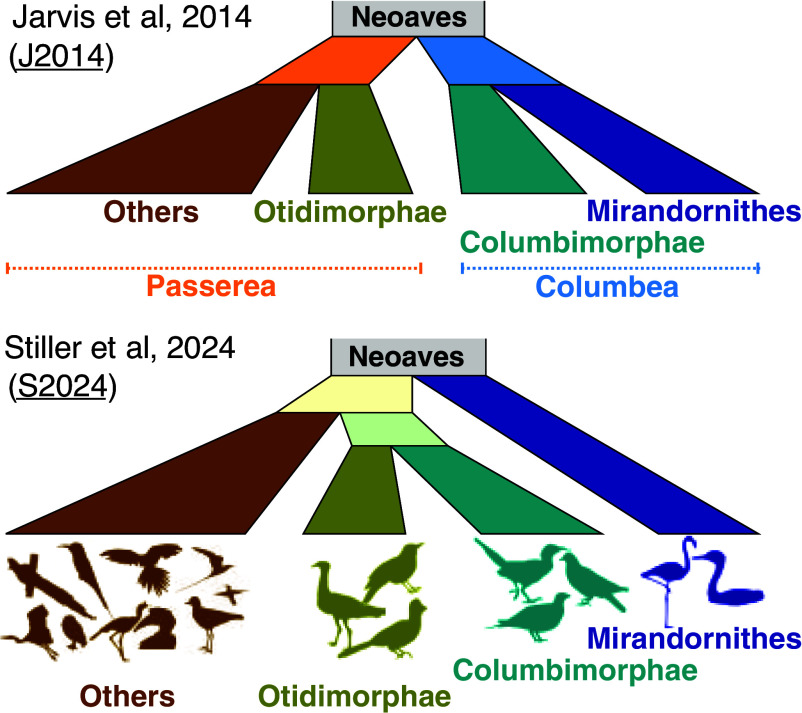
The topologies recovered by Jarvis et al. (10) (J2014) using 48 species and Stiller et al. (21) using 363 species (S2024). J2014 splits Neoaves into Columbea (Columbimorphae and Mirandornithes) and Passerea (all other Neoaves). S2024 places Mirandornithes as the sister to other Neoaves and puts Columbimorphae as the sister to Otidimorphae within the other Neoaves.

Among key findings by Jarvis et al. (10) was the division of Neoaves into two strongly supported clades: Columbea and Passerea (Fig. 1), a topology (called J2014 henceforth) found in their analyses dominated by noncoding DNA. Columbea comprises Columbimorphae (doves, mesites, and sandgrouse) and Mirandornithes (also called Phoenicopterimorphae; flamingos and grebes). Passerea includes all other Neoaves. The division of Neoaves into Columbea and Passerea has been the subject of intense debate (5, 25, 26, 28). The new analyses by Stiller et al. (21) recovered Mirandornithes alone as the earliest diverging Neoaves, thus breaking Columbea (Fig. 1). Columbimorphae was united with Otidimorphae as the sister to all other Neoaves except Mirandornithes. This placement of Mirandornites as sister to all other Neoaves (called the S2024 topology henceforth) has been proposed before (26, 28). It was recovered by Jarvis et al. (10) when analyses were limited to ultra-conserved element (UCE) sequence data, although later analyses of UCEs with more filtering resulted in Columbea again (29). It is remarkable that the two whole-genome-based analyses disagree on this fundamental relationship, each with strong statistical support. Unfortunately, morphological data do not provide any way to resolve this disagreement because there are essentially no characters that unite clades deep in the avian tree (27).

A plausible explanation for the conflict between Jarvis et al. (10) and Stiller et al. (21) is the impact of improved taxon sampling, though in the context of species tree estimation rather than the traditional arguments that focused on only a few genes (30). In this study, we show that while taxon sampling plays a role, what makes it especially relevant in this case is the existence of a striking outlier region of a single chromosome (Chr 4 in chicken). The locus trees in this region (21 Mb long; Table 1) show uncharacteristically low levels of discordance and consistently support J2014. This is in profound contrast to the rest of the genome that shows abundant and stochastic discordance with frequent changes in the topology, as expected under ILS; genome-wide analyses, on aggregate, support the S2024 topology as the species tree. Our results suggest that there was a period around the early diversification of Neoaves when recombination was strongly suppressed in the chromosome 4 outlier region across more than one speciation event. Remarkably, the strong phylogenetic signal of that event has persisted in extant genomes. These patterns dramatically diverge from ILS expectations based on the rest of the genome and require invoking more complex processes.

**Table 1.** Outlier regions in chr. 4 according to coordinates of the chicken genome assembly versions GalGal4 and GalGal6 (shown parenthetically)

Start coordinate	End	Length	# Loci
25030k (25555k)	32680k (33202k)	7.64 Mbp	535
33510k (34230k)	34480k (34999k)	0.96 Mbp	48
44130k (44690k)	56820k (57179k)	12.68 Mbp	848

Number of locus trees in a region is shown.

## Results

### Unexpected Discordance-Free Signal Supporting Columbea in a 21-Mb Region.

**Fig. 2.**
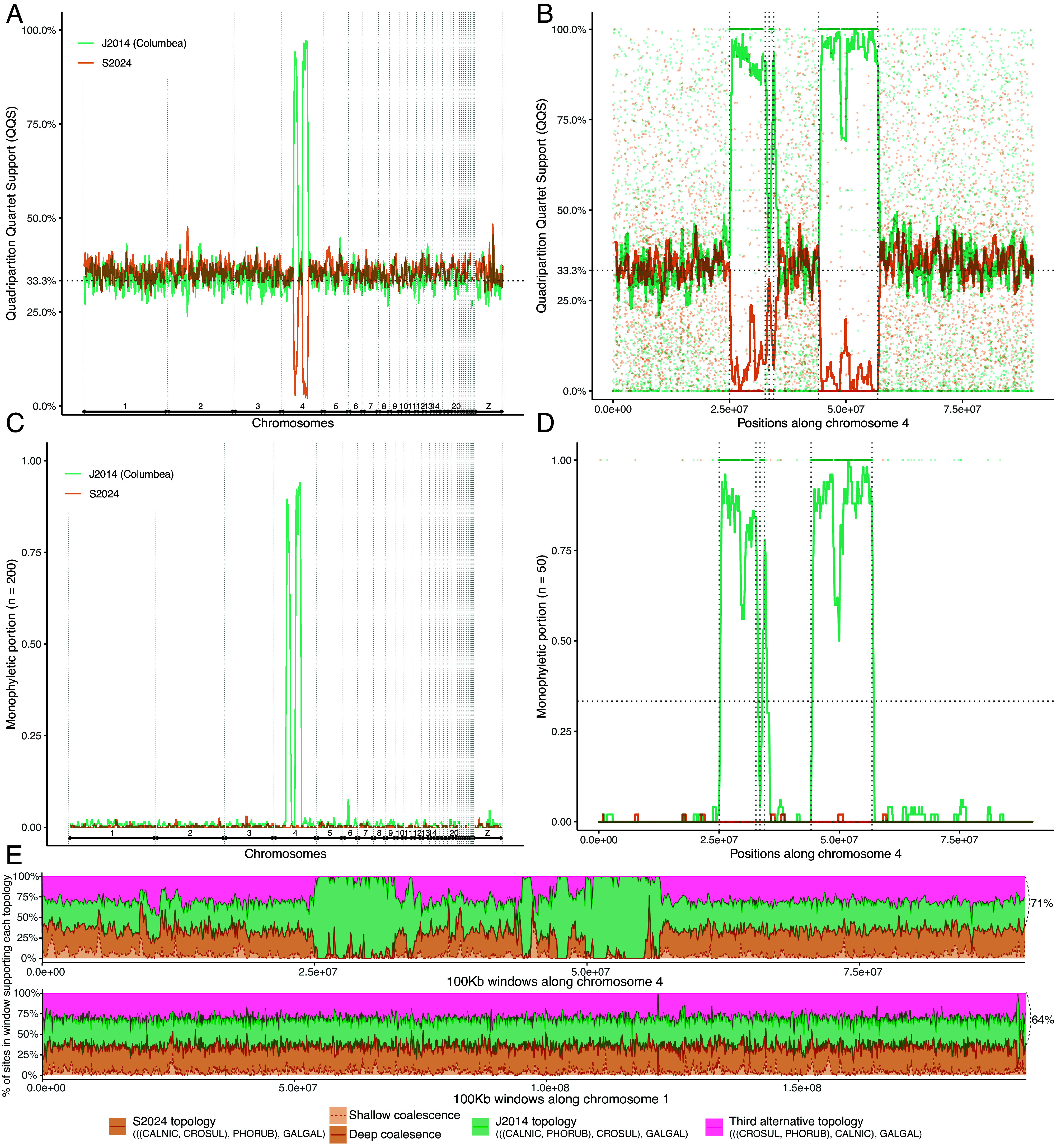
Strong signals within chromosome 4. (*A*) The moving average (over 200 consecutive loci) of the QQS measure of support for branches representing the J2014 topology (Columbea), and the S2024 topology across all chromosomes (delimited by vertical lines). (*B*) Similar moving average (over 50 consecutive loci) for chromosome 4 only and showing a dot for each locus. QQS is the proportion of the quartet trees induced by each locus tree that are in agreement with each branch examined; branches are encoded as quadripartitions as detailed in *Materials and Methods*. (*C* and *D*) Similar to A and B respectively, except support is measured as whether a locus recovers Columbimorphae and its sister (according to S2024 or J2014; see Fig. 1) as mutually monophyletic (encoded as 1) or not (0); thus, the moving average shows the percent of preceding 200 loci that recover a clade as monophyletic. Vertical dotted lines in (*B* and *D*) delineate the areas with extremely high support for Columbea and Columbimorphae. (*E*) Using CoalHMM across chromosomes 4 and 1. Focusing on four species, we assume S2024 as the species tree and use CoalHMM to compute support for each of the three possible quartet topologies, distinguished by color. We show the posterior probability of topologies, computed over 100 kb regions; see *Materials and Methods* for details. For the species tree topology (S2024), the same topology can be recovered with deep or shallow coalescence, distinguished here. The total probabilities of topologies other than S2024 give an estimate of the amount of ILS and are show on the right (ILS levels $>\frac{2}{3}$ violate the ILS-only MSC model). Outlier regions in chromosome 4 show a dearth of discordance among loci.

We first interrogated the genome-wide signal of several challenging branches using 63,430 intergenic locus trees generated by Stiller et al. (21). We quantified the support for 16 hypothesized branches in each genomic region using a measure called quadripartition quartet support (QQS) (*Materials and Methods*). We examine all 13 nodes among the early radiation of Neoaves identified by Stiller et al. (21) as having high-ILS (defined as weighted mean of QQS <0.37), Columbea, and two controversial nodes among Palaeognathae (*SI Appendix*, Fig. S1*A*). For these challenging nodes, QQS averaged over consecutive loci showed a relatively stable pattern, with a major exception (*SI Appendix*, Figs. S1*B* and S2). Across three nearby regions (Table 1) of chromosome 4 (two of which are very close according to the chicken coordinates) with a total length of ≈21 Mb, there was a drastic reduction of support for the S2024 topology and an extremely high level of QQS for the J2014 topology (Fig. 2 *A* and *B*). No other region in the genome and no other high-ILS node showed anything similar to these regions in terms of strong support for one of the alternative topologies across extended regions (*SI Appendix*, Fig. S1*B*). We will refer to these coordinates of chromosome 4 as “outlier regions” henceforth. Examination of six Neoavian exemplar genomes with high-quality chromosomal level assemblies from Vertebrate Genome Project (VGP) (31) revealed that these outlier loci map to a single contiguous region of the chromosome 4 homolog in several species, often located at one chromosome end (*SI Appendix*, Fig. S3*A*). Similar patterns were observed (*SI Appendix*, Fig. S4*A-C*) when we examined another measure of quartet support called BQS focused on a single branch (*Materials and Methods*), as opposed to a quadripartition, formed from a branch and its four adjacent branches.

**Fig. 3.**
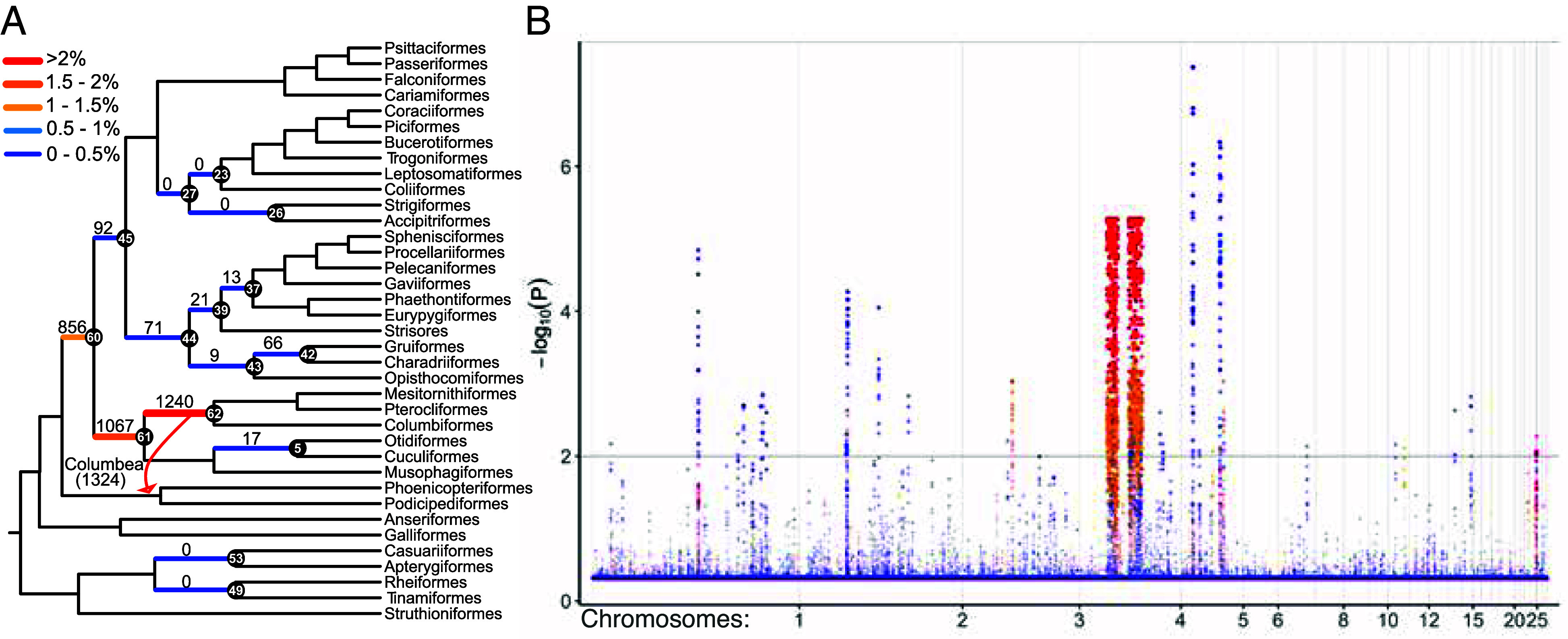
(*A*) The S2024 cladograms, marking in black and labeling (in white) 14 branches identified by Stiller et al. as having very high ILS, in addition to one controversial medium ILS branch (node 49). For these 15 branches and the Columbea branch of J2014 (red arrow), we use quartet frequencies to statistically test (*Materials and Methods*) whether each sliding window of 20 consecutive loci (i.e., ≈ 200 Kbp) supports the branch at levels that fall outside of the normal range for that node established using all ≈55,000 windows. Branch labels and colors show the number and percentage of windows that support each branch at unexpected levels, defined as a P-value <0.01 after Benjamini–Hochberg (BH) multiple testing correction. (*B*) A Manhattan plot, showing the $\log_{10}$p-values for each window and each identified branch, coloring branches similarly to (*A*). Only chromosomes 1 to 28 are included; we excluded sex chromosomes because they are expected to have atypical coalescent histories.

To formally test whether the strong support for one topology in long stretches of the outlier region is unexpected under the MSC model of ILS, we devised a statistical test (*Materials and Methods*). Our test uses the observation, rooted in the MSC theory (32), that QQS averaged over sufficiently many loci (here, 20 consecutive loci, corresponding to roughly a 200 Kb region) follows a normal distribution concentrated around the genome-wide mean (*SI Appendix*, Fig. S5*A–C*); we simply quantify the deviations from this expectation to obtain a P-value for each window of 20 loci. Consistent rejection of the null hypothesis in a region would indicate that it does not follow the MSC model. Our results confirm clearly that the outlier region stands out for the focal branches. For most examined branches of the tree, very few windows (often zero and <100 windows in every case) reject the MSC model (Fig. 3*A*). In contrast, for branches that distinguish J2014 and S2024 and those adjacent to them, between 856 and 1,324 windows rejected the MSC model (see *Materials and Methods* as to why adjacent branches are impacted by QQS), and these windows fall in the outlier region (Fig. 3*B*). For branches unrelated to the differences between J2014 and S2024, the small number of windows that reject the MSC did not form long and contiguous regions as the outlier regions.

We next asked whether support for Columbea outside the outlier region is quantitatively different than within that region. Most locus trees failed to resolve high-ILS clades as monophyletic (*SI Appendix*, Fig. S6). The outlier region on chromosome 4 was an exception; the vast majority of the locus trees in this region consistently found Columbea as monophyletic with high support (Fig. 2 *C* and *D*). Out of 1,431 loci in the outlier region, 1,375 included at least one taxon from Columbimorphae, Mirandornithes, and Passerea; among these, 1,197 (87%) recovered Columbea. Although we expect some locus trees to include Columbea by chance alone, we only found 372 loci outside the outlier region (with the same taxon requirements) that recovered Columbea (despite 50 times more sequence data than the outlier region), and these were distributed across the genome (*SI Appendix*, Fig. S7*A*). Moreover, among locus trees that recovered Columbea, the branch uniting Columbea was on average twice as long among those in the outlier regions than those outside that region (0.0088 vs 0.0045 expected substitutions per site on average; *SI Appendix*, Fig. S7*B*). The length of the branch uniting Columbea provides information about the coalescent times; the two-fold difference in branch lengths indicates that the coalescent history for loci in the outlier region is fundamentally different from the coalescent histories for loci outside of the outlier region that recover Columbea.

To further interrogate coalescence scenarios, we used CoalHMM (7) (*Materials and Methods*), which is a hidden Markov model that runs along the sequence alignment and directly estimates shifts in locus tree topologies due to ancestral recombination, making it more robust to recombination. This analysis also showed a strong signal of discordance-free support for Columbea, incompatible with MSC, in the outlier region of chromosome 4 (Fig. 2*E*). Assuming the S2024 topology, chromosome 1 experienced high levels of ILS (64% of positions disagreeing with the species tree) but followed the MSC expectations. In contrast, the outlier region showed exclusive support for Columbea, in ways that are not consistent with the MSC. This region would have to have 71% quartet disagreement with the species tree, which is >2/3 and not admissible under the MSC model. The CoalHMM results provide additional evidence that these outlier regions, unlike the rest of the genome, have experienced very little recombination among branches where Mirandornithes, Columbimorphae, and Otidimorphae were diverging ca. 66 Mya (21). Thus, the evolutionary history of this region is uncharacteristically homogeneous compared to other regions of the genome.

### Ruling Out Artifactual Causes.

We examined whether the high support for Columbea in the outlier regions can be attributed to analytical factors such as including more informative sites, biases in the evolutionary model, or rate variation. No such evidence was found (*SI Appendix*, Fig. S8). Loci in the outlier regions did not show any discernible difference with the rest of the genome in the number of species included (and thus missing data levels), branch length properties (e.g., length, stemness, clocklikeness), branch support, GC composition, or portion of informative sites. The outlier regions were also not different in terms of the presence of protein-coding genes compared to the rest of chromosome 4; the outlier regions include 23.6% of the total length and 21.3% of the genes (*SI Appendix*, Fig. S3*B*).

We also examined the effect of model misspecification using two approaches: analyses using Lie Markov models, which allow nonhomogeneous base frequencies across the tree (33) and RY coding, which reduces the impact of variation in GC-content (28). Because of its computational cost, we used Lie models to analyze only 10 randomly selected outlier loci. All of those analyses still recovered Columbea, suggesting that nonhomogeneous patterns of sequence evolution were not the cause of recovering Columbea. We applied the less computationally demanding RY coding analysis to 1,500 loci, selecting at random 500 loci from the outlier region and 1,000 from the rest of the genome. RY encoding only slightly reduced QQS for Columbea both in the outlier region (from 97.6 to 86.3%) and outside of the outlier region (from 35.4 to 33.8%), highlighting that base composition does not explain the differential recovery of Columbea. Thus, the patterns observed cannot be attributed to artifacts of inferences and are likely due to biological processes.

### Outlier Regions Interact with Taxon Sampling to Impact the Inferred Species Tree.

**Fig. 4.**
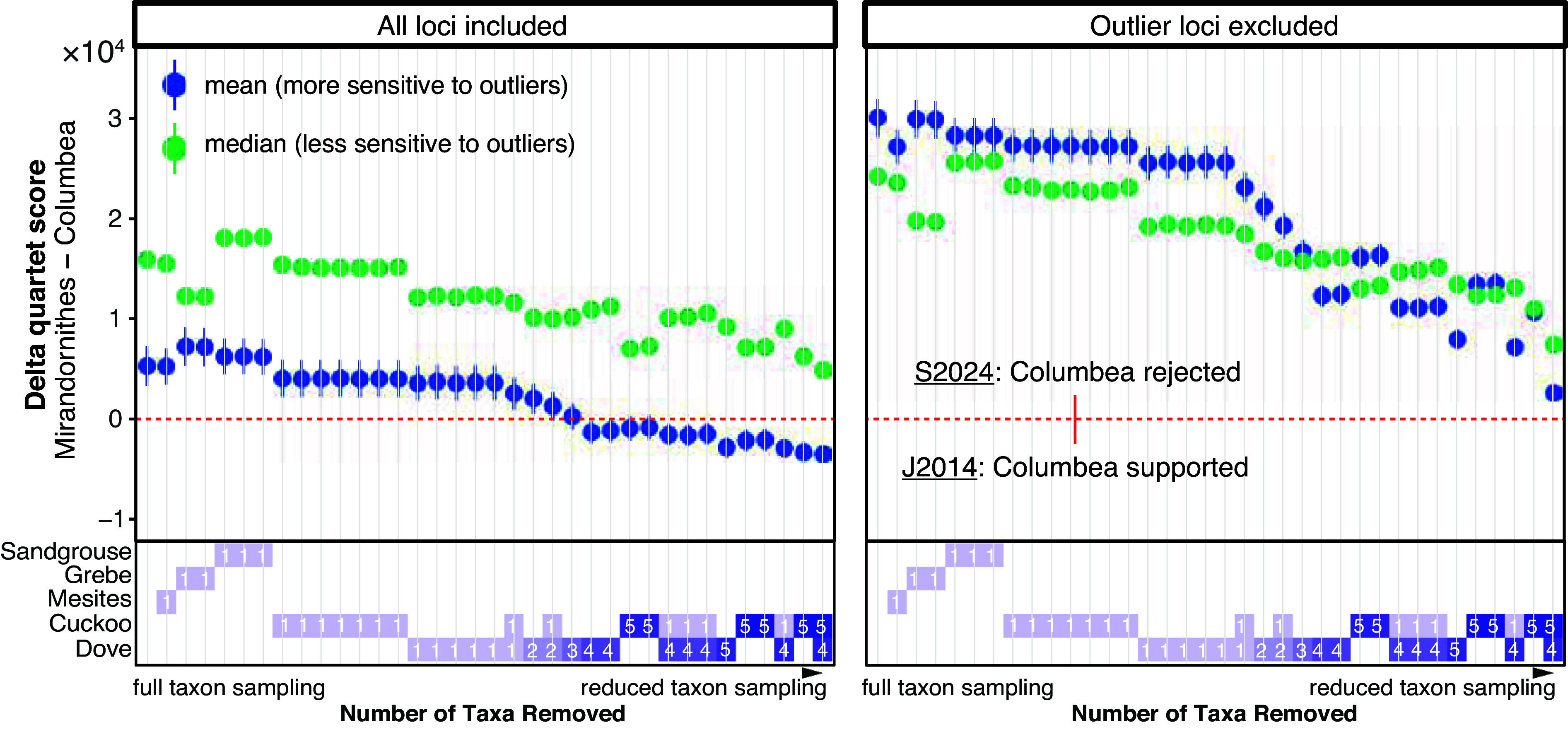
Support for the S2024 topology relative to support for J2014 for various taxon samples with and without the outlier regions. Note that the S2024 topology is always favored unless taxon sampling is reduced and the outlier regions of chromosome 4 are included (mean support above zero indicates that ASTRAL will recover S2024). y-axis: the difference between the quartet score of S2024 and J2014 trees, showing median, mean, and SE over 63,430 locus trees from the S2024 study. x-axis: the numbers of taxa (1 to 6) removed from S2024 locus trees for each group shown as a heatmap. Owing to the presence of outliers, the median (which is more robust to outliers) and mean (sensitive to outliers) diverge. Reducing the number of doves and cuckoos reduces support for S2024 compared to J2014, eventually leading to the recovery of J2014 when at least four of the five doves or five out of the seven cuckoos are removed. Excluding the outlier region of chromosome 4 makes the median and mean similar and leads to the S2024 regardless of taxon sampling.

Although the outlier regions make up only 2% of the total loci, their inclusion or exclusion strongly impacted the resolution of early Neoaves divergences inferred by ASTRAL (*SI Appendix*, Fig. S9). Applying ASTRAL to all the 63,430 intergenic locus trees of Stiller et al. (21) but restricted to the 48 species studied by Jarvis et al. (10) recovered Columbea, just as in the J2014 topology (*SI Appendix*, Fig. S9*C*). However, removing the outlier regions from these 48-taxon locus trees resulted in a topology very similar to S2024 but with Otidimorphae as sister to doves, breaking Columbimorphae (*SI Appendix*, Fig. S9*D*). The rest of the tree did not change after removing outlier regions. With the increased taxon sampling of Stiller et al. (21), the S2024 topology was recovered regardless of whether the loci in the outlier regions were included. Consistent with this observation, reducing the taxon sampling gradually reduced the support for the S2024 topology (Fig. 4). However, without the outlier region, the S2024 topology was recovered regardless of the taxon sampling. Thus, both lowered taxon sampling and the inclusion of the outlier region biased analyses toward the J2014 topology, which is recovered only when both sources of bias are present.

Reddy et al. (5) recovered the J2014 topology using only 54 loci. All analyses in that study had ≥ 95% bootstrap support for Columbea, a surprising result given that other studies based on similar numbers of loci (34, 35) were unable to recover any support at the base of Neoaves. Here, we provide an explanation for the earlier result; Reddy et al. (5) included the PPP2CB locus, which is in the outlier region. In fact, an analysis of a single intron in PPP2CB had ≥75% support for Columbea (20). Thus, it seems some of the conflicting signals observed for this relationship in prior phylogenomic studies can be traced to loci located in the outlier region.

### Examining Causes of the Outlier Regions.

The results indicated a lack of recombination in this region for an extended period of time when Neoaves diversified ca. 66 Mya (21). The fact that the recovered species tree with increased taxon sampling, or with lower taxon sampling and excluding the outlier region, both recover the S2024 topology is evidence that it is likely the correct species tree. Regardless of the species tree, explaining the strong signal for the J2014 topology across long stretches of the genome requires explanations beyond MSC. We put forward two hypotheses.

#### Rearrangement hypothesis.

**Fig. 5.**
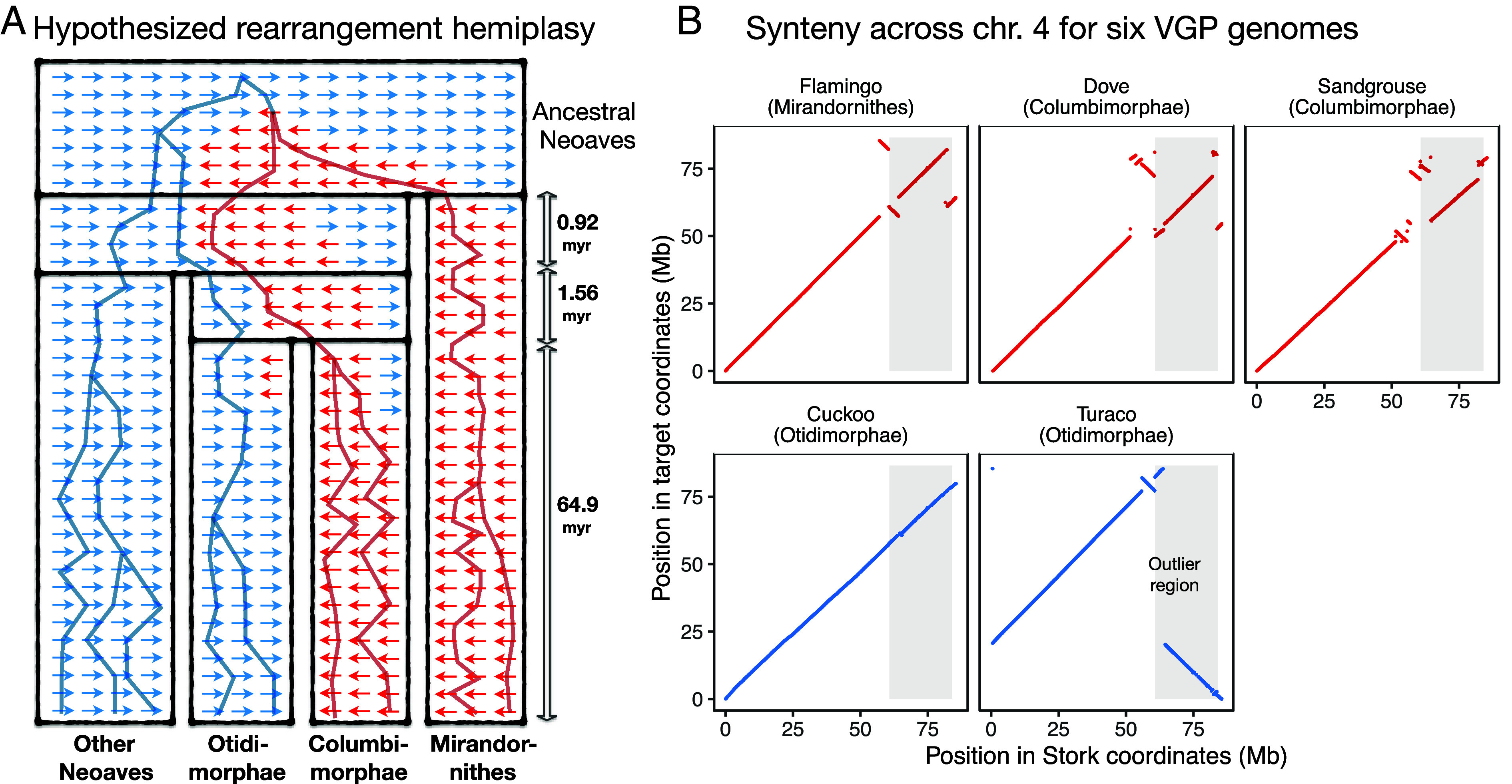
Rearrangements can explain outlier genes. (*A*) Illustration of a scenario of rearrangements that could have given rise to the observed patterns. One or more rearrangements in the common ancestor of Neoaves stayed polymorphic through two subsequent speciations, spanning at least 2.5 million years of evolution. This rearrangement created a lack of successful recombination in this region, which then led to strong sorting of locus trees in this region. Subsequently, the allelic forms were fixed with a pattern that conflicts with the speciation history. (*B*) Examining chromosome 4 of six high-quality genomes from the VGP, showing synteny of stork versus the other five. We see evidence for the proposed scenario presented in *A*. Around the boundaries of the outlier region (shaded in gray), there are rearrangement events observed in Columbea, with similar patterns. In contrast, cuckoo seems to lack such rearrangements compared to other Neoaves (represented by stork). Turaco, the other Otidimorphae, does include some rearrangement but unlike Columbea.

In this hypothesis, one or more rearrangements happen at the boundaries of the outlier region in the ancestral population of Neoaves. Considering that this species likely had a very large population size (refs. 21 and 23), we hypothesize that the rearrangement(s) persisted as a polymorphism through the rest of the lifetime of this ancestral species in addition to two subsequent speciation events (Fig. 5*A*). This amounts to maintained polymorphism of a rearrangement for at least 2.5 million years according to the dated tree of Stiller et al. (21); such an event has a 25% chance, assuming a generation time of 5 y and a population size of 250,000. The large-scale rearrangement presumably prevented or dampened recombination in individuals that were heterozygous for the rearranged region, which, despite their size, would behave as a pair of alleles in the ancestral population (we refer to the alternative forms of rearranged regions as allelic forms to reflect their size and the possibility that recombination was reduced but not completely eliminated.) The rearrangement(s) would then be sorted such that one allelic form is fixed in the ancestral Columbimorphae and Mirandornithes (i.e., Columbea) while the other allelic form is fixed for Otidimorphae and the rest of Neoaves (Fig. 5*A*). This scenario would lead to a large region of the genome remaining recombination-free between the two allelic forms for a substantial time period, creating a strong signal for the J2014 topology in the outlier region.

To evaluate this hypothesis, we examined synteny across six high-quality VGP genomes spanning the groups in question. We observed rearrangements at the boundaries of the outlier region, including inversions and translocations (Fig. 5*B*). The Columbea species had similar synteny patterns in and around the boundaries of the outlier region, while cuckoo (among Otidimorphae) was very similar to the exemplar genome we selected among Neoaves (Stork). The other Otidimorphae examined (Turaco) also showed signatures of a rearrangement around the boundaries of the region, but its rearrangement (a large inversion and translocation) was different from Columbea. Examining Hi-C interactions did not provide evidence of misassembly near the breakpoints in the Turaco genome (*SI Appendix*, Fig. S10). The Hi-C mapping was generally supportive of the structural accuracy of the assembly, although the presence of a sequence gap at the breakpoint boundary of the large inversion in Turaco suggests some caution. Most likely, these Turaco rearrangements are an unrelated event that happened on the branch leading to Turaco after it diverged from the Cuckoo.

Interchromosomal rearrangements in avian chromosome 4 have been previously reported (36), including in warblers where they created a neo-sex chromosome (37) (no synteny to Z was found in our analyses). The additional differences in synteny compared to our baseline scenario (Fig. 5) may be explained by subsequent rearrangements over the past 65 Myr. Nevertheless, the prevalence of rearrangements around the boundaries of the outlier regions suggests that these boundaries may exhibit a high rate of rearrangement, lending support for the rearrangement hypothesis. Thus, the synteny data are consistent with the hypothesis that polymorphic rearrangements could have suppressed recombination in the outlier region over an extended period of time.

#### Hybridization hypothesis.

Another hypothesis invokes hybridization and subsequent selection on the outlier region. This would require gene flow from an ancestral Mirandornithes population to an ancestral Columbimorphae population (*SI Appendix*, Fig. S11*A*), due to hybridization between species that had started diverging at least 2.5 Myr earlier. Gene flow would have been one-directional or else we would also see an abundance of locus trees that unite Columbea and Otidimorphae sister to all other Neoaves (which we do not see). Furthermore, hybridization alone does not explain why the outlier region is devoid of discordant topologies and why the strong signal is present in this particular region and nowhere else. Gene flow alone would predict a dispersed pattern of gene tree topologies because even in the presence of hybridization, ILS and recombination still act to create stochastic changes across short regions of the genome (38).

The hypothesis that gene flow caused the observed patterns requires one to make additional assumptions. The simplest of those assumptions would be strong selective pressure for some genes spanning this region, making it deleterious to carry the alleles not inherited from Mirandornithes in the ancestral Columbimorphae population. Although cases of adaptive introgression are documented for other species (39), they require a strong selective pressure. However, we found no evidence that the outlier genes are enriched in positive selection based on the dN/dS ratios of 992 genes located in chromosome 4 (*SI Appendix*, Fig. S11*B*). We did find evidence for modest enrichment of Gene Ontology (GO) terms related to cytokine activity (*SI Appendix*, Fig. S12). Balancing selection at cytokine loci has been documented (40, 41), so introgression of a rearranged region might be favored. Alternatively, balancing selection could have favored retention of a polymorphic rearrangement for an extended period of time. In either case, finding evidence of selection that occurred 65 Mya is difficult, and it is not necessary for many genes in the region to have been under selection. In principle, two genes at the boundaries would suffice if we assume that the rearrangements observed in this region are independent of the selection (Fig. 5*B*). A single gene might suffice if we assume the rearrangements occurred in the ancestor of Mirandornithes and recombination was suppressed upon introgression in the ancestral Columbimorphae. Although we cannot fully reject the hybridization+selection hypothesis, it does require assuming selection given the absence of clear evidence for introgression elsewhere in the genome. This makes the simpler rearrangement+ILS hypothesis more likely.

## Discussion

Our results have several implications for future studies. Such strong signals of depleted discordance relatively deep into evolutionary history spanning a large contiguous region have not been documented before to our knowledge. The presence of individual outlier genes with a large impact on the species tree has been documented (35, 42), but our observations are different. They reveal a large region with high support and low discordance for a particular topology that is different from the species tree. We were only able to identify this signal because Stiller et al. (21) built trees from windows selected across the genome, allowing us to look for positional signals. Similar analyses should be performed for other organisms, a task that will only be helped in the near future as new high-quality genomes become available. Moreover, our analyses focused on ILS and discordance among gene trees. We note that Jarvis et al. (10) recovered Columbea using concatenation as well, showing that it can also be sensitive to these outlier loci, a topic that can be further explored in the future.

Our results superficially resemble the century-old concept of supergenes, a long region of the genome encompassing multiple genes that has experienced recombination suppression and contributes to a specific phenotype effect (43, 44). Supergenes are widely studied across diverse groups of species, including birds (45, 47). Chromosomal rearrangements in general and inversions, in particular, have been implicated in supergene formation (43), especially in recent analyses (46, 48, 49) and occasionally together with subsequent introgression of inversions (50). Supergenes differ from our results in a crucial way: Past analyses of supergenes have focused on diversity within species, at population genetic scales, spanning hundreds of thousands or at most a few million years of evolution. What we have documented is at a deep phylogenetic level across present-day orders. These outlier regions could have been similar to present-day supergenes 65 Mya. However, we note that while the standard definition of supergenes is based on phenotype and function, the phenotypic significance of the outlier region we have detected is unclear, making us hesitant to call the region a supergene.

More broadly, our results hint at the often ignored possibility of ILS mediated by rearrangements persisting through long evolutionary times. As previously argued, rearrangements can provide strong signals for recovering phylogenetic relationships (51). However, less appreciated is that rearrangements can be subject to ILS. Surprisingly, the analytical methods used in some of the earliest reconstructions using rearrangement (52) implicitly minimized the number of branches over which polymorphic rearrangements must be maintained (53). Our study shows that the poorly appreciated interplay between ILS and rearrangements can have a major impact in modern phylogenomic studies. The presence of such long stretches contradicts the MSC theory behind most species tree inference methods and will have implications for how to select loci across complete genomes. Future studies would benefit from advanced theoretical and empirical investigations of how rearrangements can mediate ILS. As more high-quality genomes become available, many of the questions in phylogenomics should be revisited with an eye on the interaction between rearrangements, ILS, and other sources of discordance such as hybridization.

## Materials and Methods

### Quantifying Support for Specific Branches.

We measure support for each branch of the species tree using four metrics.

#### Quadripartition quartet support (QQS).

An internal branch of an unrooted tree along with its four adjacent branches defines a quadripartition of taxa, denoted by A·B∣C·D. For example, the branch uniting Columbea as the sister to Passerea (J2014 topology) has the quadripartition: Columbimorphae·Mirandornithes∣Passerea·Non-neoaves. For a fully resolved locus tree t and a quadripartition A·B∣C·D, we define QQS to be the portion of quartets of taxa with exactly one taxon selected from each of A, B, C, D that display a topology in t consistent with A·B∣C·D. More precisely, for a∈A,b∈B,c∈C,d∈D, the quartet a,b,c,d is consistent in locus tree t with quadripartition A·B∣C·D if and only if t restricted to the quartet has the unrooted topology ab∣cd. By convention, we let the outgroups be part of partition D. Then, it becomes clear that the QQS for A·B∣C·D is effectively evaluating support for the mutual monophyly of A and B. Each quartet in a resolved locus tree is consistent with either A·B∣C·D, A·C∣B·D, or A·D∣B·C. Thus, we can normalize the number of quartets supporting each quartet by the total number of quartets, obtaining a normalized QQS for that branch such that the QQS of the three alternative topologies add up to one (54). A QQS of 1/3 corresponds to a polytomy (55).

Here, we report normalized QQS for Columbea (noted above) and fourteen quadripartitions selected from the S2024 tree that correspond to high-ILS nodes (21), defined as those with QQS less than 0.37 after collapsing low support branches (<0.95) in the gene trees (*SI Appendix*, Fig. S1*B*). We also include the quadripartition Rheiformes·Tinamiformes∣Apterygiformes+Casuariiformes·Other-birds, which had QQS 0.39 but nevertheless was uncertain in the original study. We show the moving average of QQS across consecutive loci of each chromosome. We recompute and report QQS here based on fully resolved gene trees (without contraction).

#### Branch quartet support (BQS).

A single branch of an unrooted tree defines a bipartition of taxa into two groups, denoted by X∣Y. Each clade X of a rooted tree with taxon set L similarly defines the bipartition X∣L−X. For example, the Columbea clade (J2014 topology) has the bipartition: Columbea∣other-birds. For a fully resolved locus tree t and a bipartition X∣Y, we define BQS to be the portion of quartets of taxa with exactly two taxa from X and two taxa from Y that display a topology in t consistent with X∣Y. More precisely, if a,b∈X and c,d∈Y, the quartet a,b,c,d is consistent for locus tree t with bipartition X∣Y if and only if t restricted to the quartet has the unrooted topology ab∣cd. Note that (unlike the QQS score) not all considered quartets provide strong support for X∣Y. For example, for Columbea, a quartet with two Passeriformes and two Columbimorphae will be counted, even though such a quartet would have a very low chance of conflicting with Columbea. Because QQS also counts such “trivial” quartets and the number of such quartets changes across branches, the BQS measure cannot be compared across different hypothesized species tree clades. However, for a fixed clade, it can be compared across loci with similar levels of taxon sampling. We report BQS because, compared to QQS, it has the advantage of relying on only one clade and not on the adjacent branches.

#### Monophyly analyses.

A clade is called monophyletic in a rooted locus tree if the common ancestor of the group only includes species from that group that are present in that locus tree (ignoring missing taxa). For each of the 16 quadripartitions used in the QQS analyses (e.g., A·B∣C·D), we included a locus tree in the monophyly analysis only if it includes one species from each side of the quadripartition (e.g., A, B, C, and D). It is easy to see that monophyly corresponds to cases where QQS is exactly 1. To build the moving average of monophyly, we encoded each locus that recovers a clade as monophyletic as 1 and other loci as 0. We then computed the moving average of these 0 and 1 encodings among 200 consecutive genes moving along the chromosomes from the lowest position (according to chicken) to the highest. Thus, the moving average shown in the figure is the percentage of 200 locus trees preceding each (chicken) position that recover the clade as monophyletic.

#### CoalHMM analyses.

We used CoalHMM (7) to determine whether intralocus recombination had an impact on our results. CoalHMM takes long aligned regions without predefined locus boundaries as input. CoalHMM uses hidden states corresponding to possible topologies. It uses the HMM machinery to scan the region (we used 1-Mbp windows) and detect the boundaries between locus topologies. However, because the state of topologies increases rapidly with more species, it can only be run on four species. To examine the central hypothesis of this work, we selected four taxa: *Caloenas nicobarica* (Nicobar pigeon), *Crotophaga sulcirostris* (Groove-billed ani, a cuckoo), *Phoenicopterus ruber* (American flamingo), and *Gallus gallus* (chicken), with the last one used as the outgroup. This selection allows us to test the two hypothetical trees presented here.

We ran CoalHMM (56) on chromosomes 1 (selected as a control) and 4 for these four species, using an automated workflow (57). CoalHMM outputs the posterior probability of each nucleotide site belonging to each of the hidden states, which was extracted and post-processed using custom python scripts. Focusing on each quartet, we can use these probabilities to assign each site to one of four categories: shallow coalescent (S) where the species tree is guaranteed to match the locus tree, deep coalescence but the locus tree happens to match the species tree ($D_{1}$), and deep coalescence with the locus tree matching one of the two alternative topologies ($D_{2}$ or $D_{3}$). We assigned each site to the state with the maximum probability and counted the number of sites assigned to each state in each 100 kb window. Referring to these counts by the state name, for each 100 Kb window, we measure the support for the main topology as $\frac{S+D_{1}}{S+D_{1}+D_{2}+D_{3}}$, and measure support for alternatives as $\frac{D_{2}}{S+D_{1}+D_{2}+D_{3}}$ and $\frac{D_{3}}{S+D_{1}+D_{2}+D_{3}}$. For the main topology, we also distinguish shallow coalescence $\frac{S}{S+D_{1}+D_{2}+D_{3}}$ from deep coalescence $\frac{D_{1}}{S+D_{1}+D_{2}+D_{3}}$.

### Statistical Test of Windows with Unexpected QQS Scores.

Recall that for each locus tree, we compute its QQS for each focal quadripartition A·B∣C·D. Consider a set of 20 consecutive loci on the same chromosome and let $Q_{i}$ be the mean QQS of this quadripartition for the $i^{\mathrm{th}}$ such sliding window. We observed empirically that, as predicted by coalescent theory (32), $Q_{i}$ values tightly concentrate around their mean for most branches (see an example in *SI Appendix*, Fig. S5*A*). Let $\mu_{Q}$ and $\sigma_{Q}$ be the mean and the SD of QQS across the entire genome. We observed empirically that

$$Z_{i}=\frac{Q_{i}-\mu_{Q}}{\sigma_{Q}}$$

closely follows the normal distribution for typical branches (see an example in *SI Appendix*, Fig. S5*B*). Thus, we can assign a *P*-value to each window by computing $\min(F\left(Z_{i}\right),1-F\left(Z_{i}\right))$ (where F is the CDF of the normal distribution) to test the null hypothesis that QQS values in that region are drawn from the same distribution as the rest of the genome. Because these tests are performed for tens of thousands of windows, we corrected them for multiple testing using the Benjamini and Hochberg procedure (58). Confirming the assumptions of the test are appropriate, for very few windows, the null hypothesis was rejected for most branches, except for the focal branches of this study.

### Rearrangements Analyses.

We constructed a multiple genome alignment of the 57 VGP-quality bird genomes (available as of 12/10/2021; list available at https://github.com/smirarab/chr4avian/blob/master/alignment/) representing 55 avian species (chicken is present with three versions) using Progressive Cactus version 2.0.4 (59) with its default alignment parameters and GPU-acceleration enabled (60). The computation was performed using Cactus’s Workflow Description Language (WDL) interface. The alignment extraction was referenced on chicken (galgal6) and performed using UCSC Genome Browser assembly hub with HAL-tools (61). To build the guide tree for Cactus, we used the commands implemented in PHYLUCE v.1.7.1 (62) to extract 5472 ultra-conserved elements (UCE) including 1,000-bp flanking regions to both sides. These were aligned using MAFFT v.7.475 (63) and cleaned using Gblocks v.0.91b (64). We identified 1455 UCE loci present in all species. The tree was generated using concatenated maximum likelihood analysis using IQTREE2 v.2.1.3 (65) under the GTR+I+G model with 1,000 ultrafast bootstrap replicates. Since UCEs are more conserved than the rest of the genome, their branch lengths are underestimated. To correct this bias, all branch lengths of the UCE tree were multiplied by a factor of 1.877; the factor is the slope estimated by matching the UCE tree to the Stiller et al. (21) tree (34 taxa matched), computing pairwise (patristic) distances between these 34 species in the two trees, and fitting a linear model with an intercept of zero. Finally, we removed five branches in clear conflict with the established relationships recovered across several studies (10, 21), creating a polytomy of degree seven at the root of Neoaves. The polytomy allows Cactus to try all combinations given the lack of certainty.

From this alignment, we extracted the alignment of six species using hal2maf: *Gallus gallus* (chicken; 5_GalGal6), *Streptopelia turtur* (turtle dove; 2_bStrTur (66)), *Pterocles gutturalis* (yellow-throated sandgrouse; 1_bPteGut1), *Tauraco erythrolophus* (red-crested turaco; 1_bTauEry1) *Cuculus canorus *(common cuckoo; 1_bCucCan1), *Ciconia maguari* (maguari stork; 1_bCicMag1). We extracted regions mapping to chromosome CM030196.1 in stork, which shows the largest synteny with chr4 in chicken.

From the 7-way genome alignment, we extracted pairwise alignments between all of the species and stork (used as a reference) and merged consecutive blocks using MafFilter (67). Synteny blocks were extracted from the pairwise alignments using maf2synteny with default settings (68). The synteny blocks were then post-processed for analysis using a custom python script with pandas. A single chromosome of all of the species shows synteny with chromosome CM030196.1 of stork, except the sandgrouse, where it maps to chr15 and chr20. To ease comparison in Fig. 2*E*, these two sandgrouse chromosomes were manually merged. Moreover, the genomic coordinates of the syntenic chromosome of the dove (LR594554.2) were flipped to match the synteny ordering of the rest of the species.

### Selection Analyses.

To test the hypothesis that selection has acted in the outlier region at an atypical level, we used a dN/dS test. We first selected subsets of taxa relevant to our hypothesis and formed three possible topologies. T1 matches the S2024 species tree; T2 matches the J2014 species tree; T3 puts Mirandornithes as sister to Otidimorphae (*SI Appendix*, Fig. S11*B*). Among the 1,119 functional genes on chromosome 4 of chicken, 992 high-quality orthologous genes can be found in at least one species; we focus on these 992 genes. We used PAML (69) version 4.9h under the branch model to score each topology under two models: a single-ω model that fixes the dN/dS ratio across the tree and a two-ω model that has a background ω and a foreground ω value on the focal branch indicated (on *SI Appendix*, Fig. S11*B*). For each gene along chromosome 4, we computed the log-likelihood and maximum likelihood (ML) ω values under all six scenarios. For each gene, we picked the topology with the highest log likelihood with the two-ω model. We then examined the foreground ω noting that ω≫1 indicates strong positive selection. For each gene, we also used the likelihood ratio test (with $\chi^{2}$ distribution with degree of freedom 1) to test whether the two-ω model is statistically better than the simpler single-ω model. We compared the ω of the best-scoring tree in the outlier regions to genes outside the outlier region. We also compared the P-value of whether the two-ω model, supporting an increased selection on the branch in question, was favored more often in the outlier region.

### GO Enrichment Analysis of the Outlier Genes.

A total of 352 functional genes are located in the outlier region of chromosome 4 based on the gene annotation available in NCBI RefSeq (GCF_000002315.5, galGal6). By obtaining the Gene Ontology (GO) annotation information from the gprofiler_full_ggallus.name.gmt database (Version 2023-07-27) (70), we applied the enrichGO function of the R package clusterProfiler (version 4.6.2) (71) to explore any possible biological implications of the outlier genes. After performing the BH correction (58), we observed four significantly enriched GO terms for the outlier genes using the entire gene set of chicken as the background and none if we use the rest of chromosome four as the background.

### Impact of Base Composition Heterogeneity.

In order to assess whether the recovery of Columbea could be an artifact caused by similar base frequencies in Mirandornithes and Columbimorphae, we used two approaches. First, we used the nonhomogeneous Lie Markov models that explicitly incorporate base frequency heterogeneity (33). We used the -m MFP+LM option in IQTREE2 (65) to infer locus trees. Due to the computational challenge of using these models, we restricted the analyses to 10 genes selected in the outlier region that recovered Columbea as monophyletic. We also used RY-encoding, implemented as Binary (0/1) encoding, followed by model selection and maximum likelihood inference using IQTREE2. Note that RY coding reduces the state space (from four nucleotides to two letters) and has two effects: 1) it reduces the signal and 2) it eliminates the effect of GC biases. These less demanding analyses were performed for 1,000 loci randomly selected from outside of the outlier region and 500 loci among the 1,431 in the outlier region. We selected a subset of loci because reanalyzing all loci is computationally demanding, and the effects could be established with the subset selected here.

## Supplementary Material

Appendix 01 (PDF)

## Data Availability

Alignments, locus trees and species trees from Stiller2023 are available on FigShare (72). In addition, for this paper, additional data, trees, tables of statistics, and scripts for data analysis are all available under Zenodo (73).
