# Supplementary material for: A region of suppressed recombination misleads neoavian phylogenomics
Source: Proc Natl Acad Sci U S A. 2024 Apr 1;121(15):e2319506121. doi: 10.1073/pnas.2319506121 (PMC11009670; doi:10.1073/pnas.2319506121)
Supplement: Supplementary file 1 — Appendix 01 (PDF) [file pnas.2319506121.sapp.pdf]

Supporting Information for

A region of suppressed recombination misleads neoavian phylogenomics

Siavash Mirarab, Iker Rivas-Gonzalez, Shaohong Feng, Josefin Stiller, Qi Fang, Uyen Mai, Glenn Hickey, Guangji Chen, Nadolina Brajuka, Olivier Fedrigo, Giulio Formenti, Jochen B. W. Wolf, Kerstin Howe, Agostinho Antunes, Mikkel H. Schierup, Benedict Paten, Erich D. Jarvis, Guojie Zhang and Edward L. Braun

Siavash Mirarab.
E-mail: smirarab@ucsd.edu

This PDF file includes:

Figs. S1 to S12
SI References

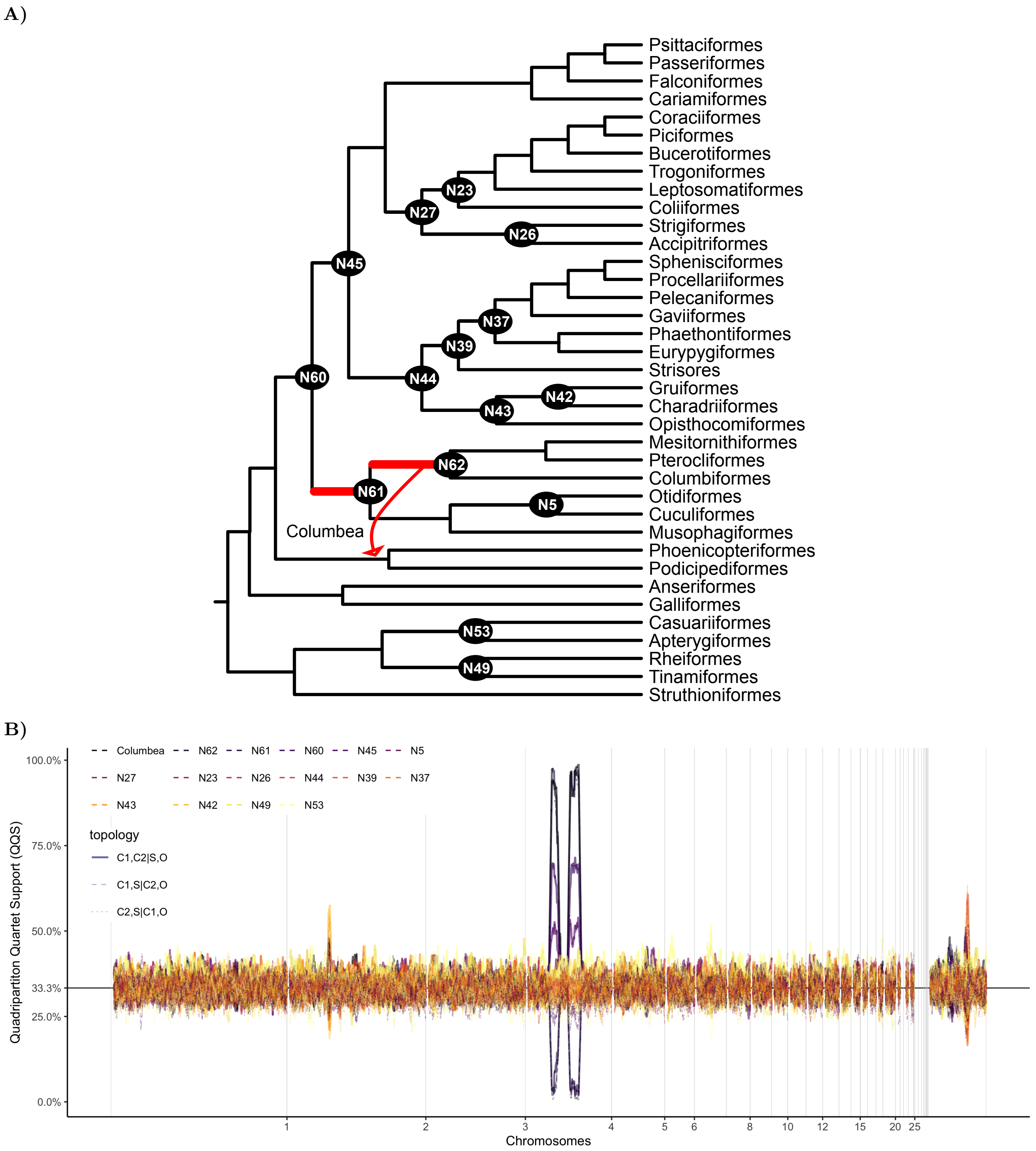

Fig. S1. A) The S2024 tree topology collapsed to orders. We mark 16 branches examined throughout the paper. All but N49 had QQS < 0.37. N49 was controversial despite having QQS=0.39. The branch labels starting with an "N" correspond to nodes of the S2024 topology using the same labels as **?**). Columbea (marked in red) is an alternative to N60 and N61, representing the J2014 topology. B) The moving average of QQS for 200 consecutive loci across different chromosomes for the 16 nodes from (A). QQS is the proportion of the quartet trees induced from each locus tree that are in agreement with each branch examined. We show QQS for the main topology ($C1 \cdot C1 \mid S \cdot O$ in solid lines) as well as the two alternatives (dotted and dashed lines). The larger chromosomes are labeled. All branches have stable QQS over 200 loci, except for the focal nodes of this study (Columbea, N60 and N61), and their adjacent nodes (N62 and N45). The definition of QQS, used in several analyses, creates dependencies among adjacent branches (see Methods). See Fig. S2 for a figure separating branches into different panels.

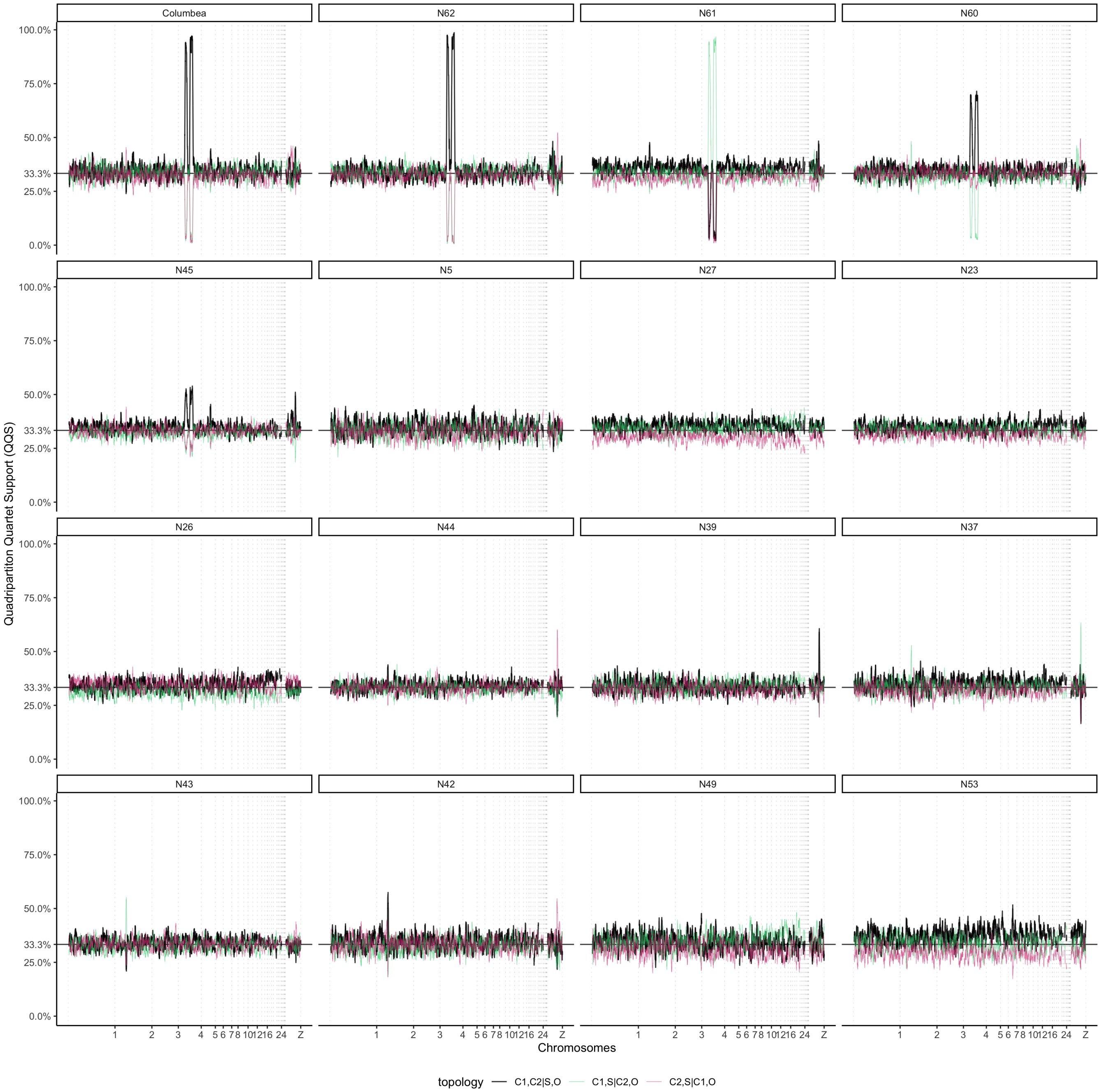

Fig. S2. The moving average of QQS for 200 consecutive loci across different chromosomes for 16 potential branches (panels), encoded as quadripartitions. All settings and labeling follow Fig. S1. The main topology ($C1 \cdot C1 \mid S \cdot O$) in shown in black and the two alternative topologies are shown in red and green.

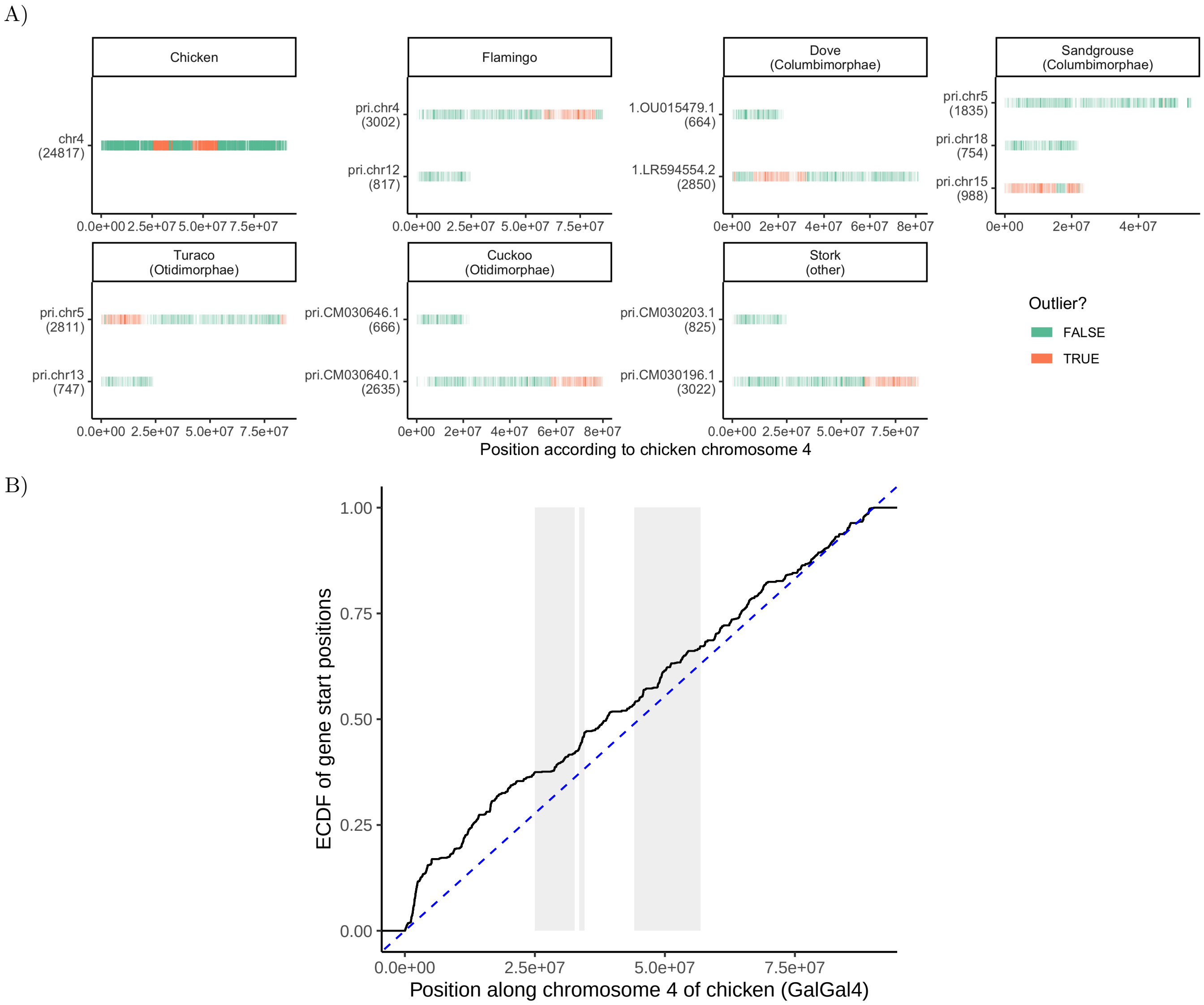

Fig. S3. A) The outlier regions fall mostly on one end of chromosome 4 in various high-quality assemblies obtained from VGP (with a few exceptions). For each of the 24817 blocks obtained from maf2synteny ($>$5000 bp) mapping to chromosome 4 of chicken (galgal6), we show the corresponding blocks in other exemplar VGP genomes, using colors to distinguish outlier regions. All chromosomes with at least 3 blocks are shown. The number of > 5000 bp maf2synteny blocks including chicken chromosome 4 and each chromosome of other species is shown parenthetically below the chromosome names. B) The empirical cumulative distribution function (ECDF), showing the distribution of genes across chromosome 4 of chicken (galgal4). The outlier regions are shown in grey. With the exception of the part of the outlier region close to bp 2.5e+07, which seems to have few genes, the outlier regions do not appear unusual relative to the remainder of chromosome 4. Overall, the outlier regions make up 23.6% of length and 21.3% of the genes, making the region unremarkable.

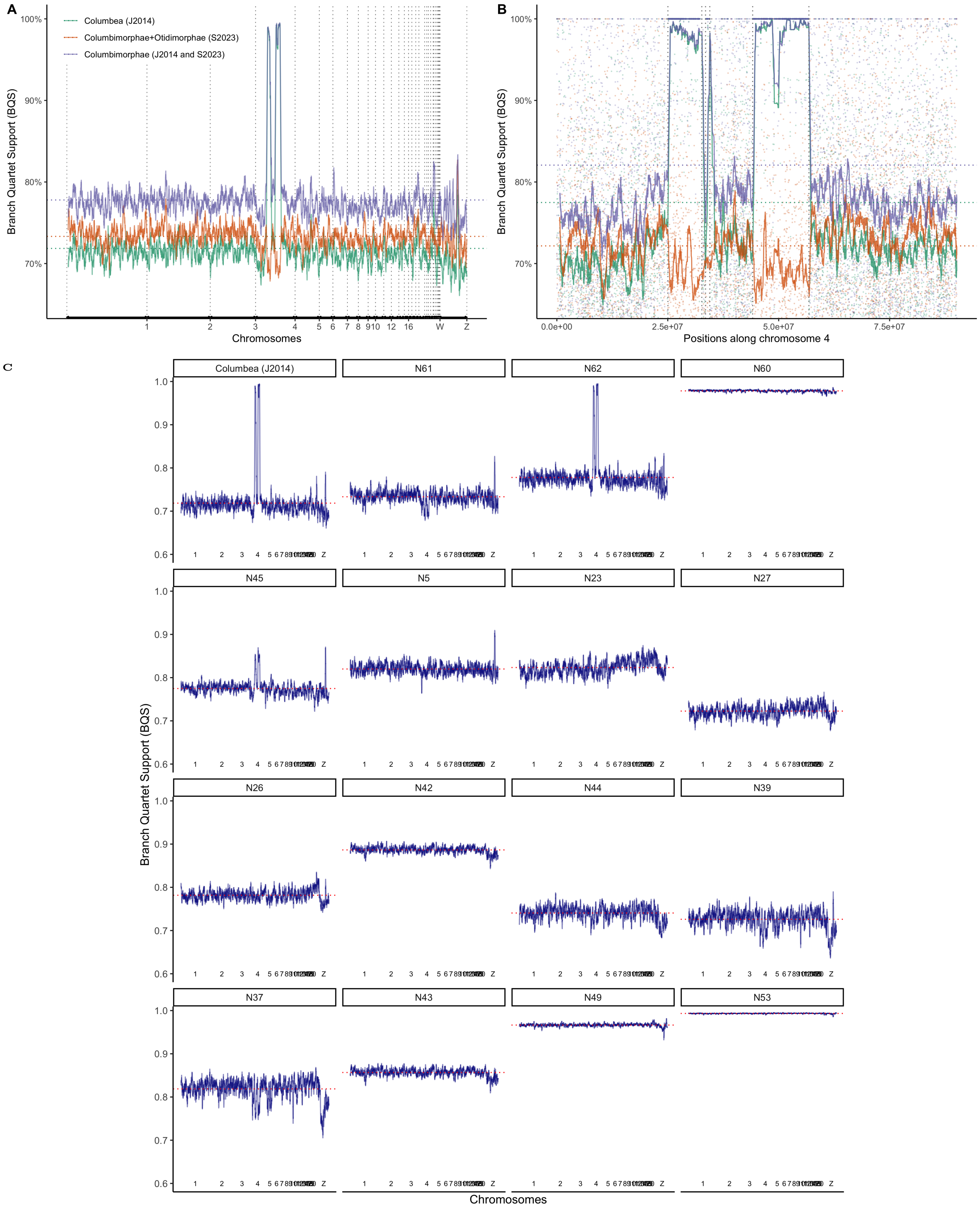

Fig. S4. The moving average of BQS for 200 consecutive loci across different chromosomes. A) The focal clades, across all chromosomes, B) focal clades focusing on Chromosome 4, C) 16 clades selected across the tree, as labeled in Figure 1C (panels). BQS is defined as the proportion of the quartet trees induced from each locus tree inferred from individual loci across the genomes that are in agreement with each clade examined; only quartets with two species within the clade and two species outside the clade are relevant to that branch and are counted here in BQS. Note that BQS cannot be compared across branches. It can only be compared across loci.

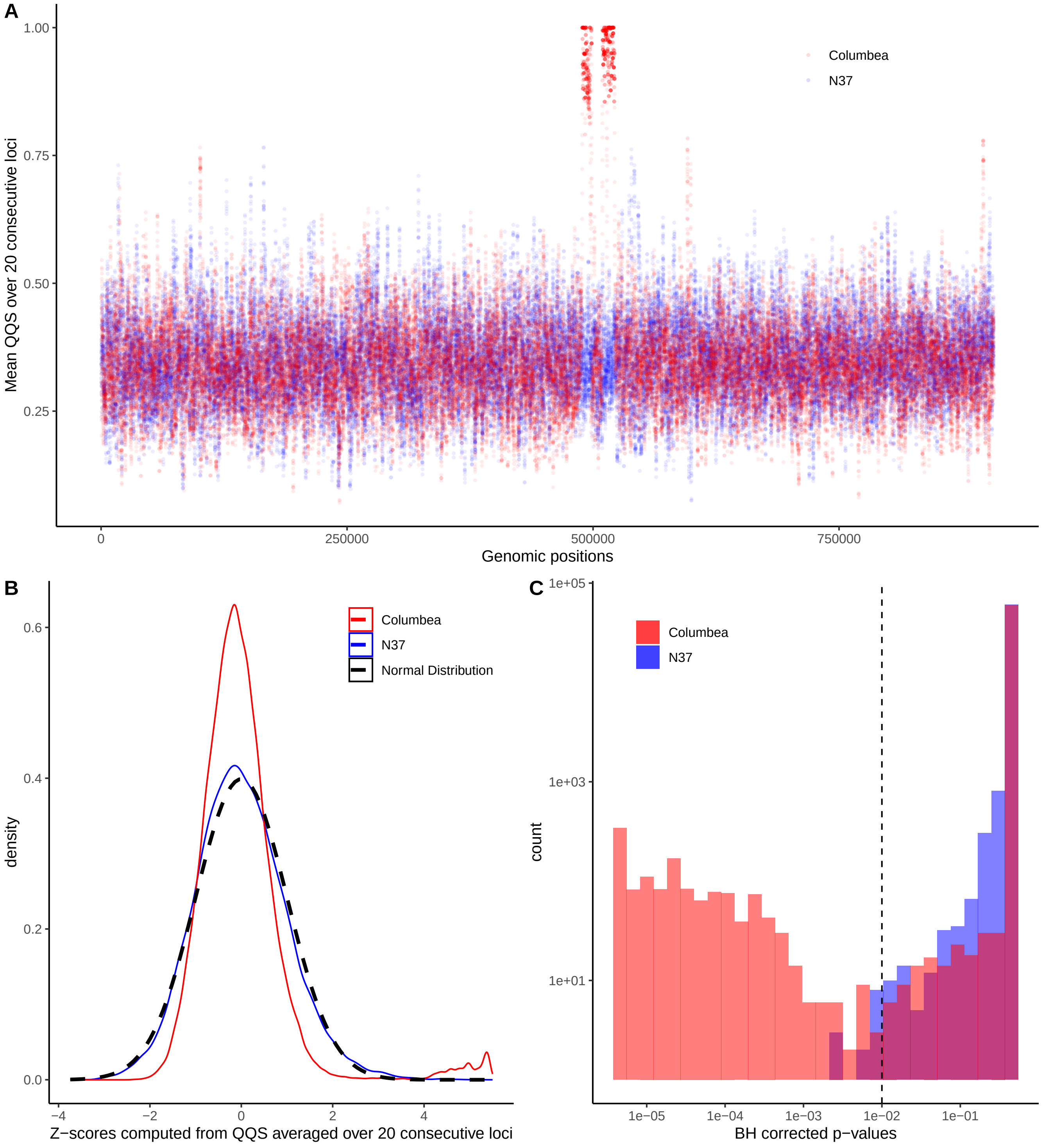

Fig. S5. A) Mean QQS computed for every 20 consecutive loci (QQS-20) shown as a dot for each sliding window of size 20. We show two quadripartitions: Columbea (Columbimorphae·Mirandornithes|Passerea·Non-Neoaves) and N37 (Aequornithes·Phaethontimorphae|Strisores·Other-birds). The former is one with apparent outliers while the latter represent an example node with normal behaviour across the genome. B) Normalizing the QQS-20 scores provides a Z-score that follows the normal distribution for typical branches (e.g., N37). Columbea clearly has a second tail of high-QQS windows. C) Using Z-scores to compute a p-value based on the cumulative density function of the normal distribution in a two-sided manner (testing for unexpectedly high or unexpectedly low QQS). Resulting p-values are corrected for multiple testing using the Benjamini and Hochberg procedure (i.e., controlling FDR). Very few outliers are found for N37, while many are detected for Columbea.

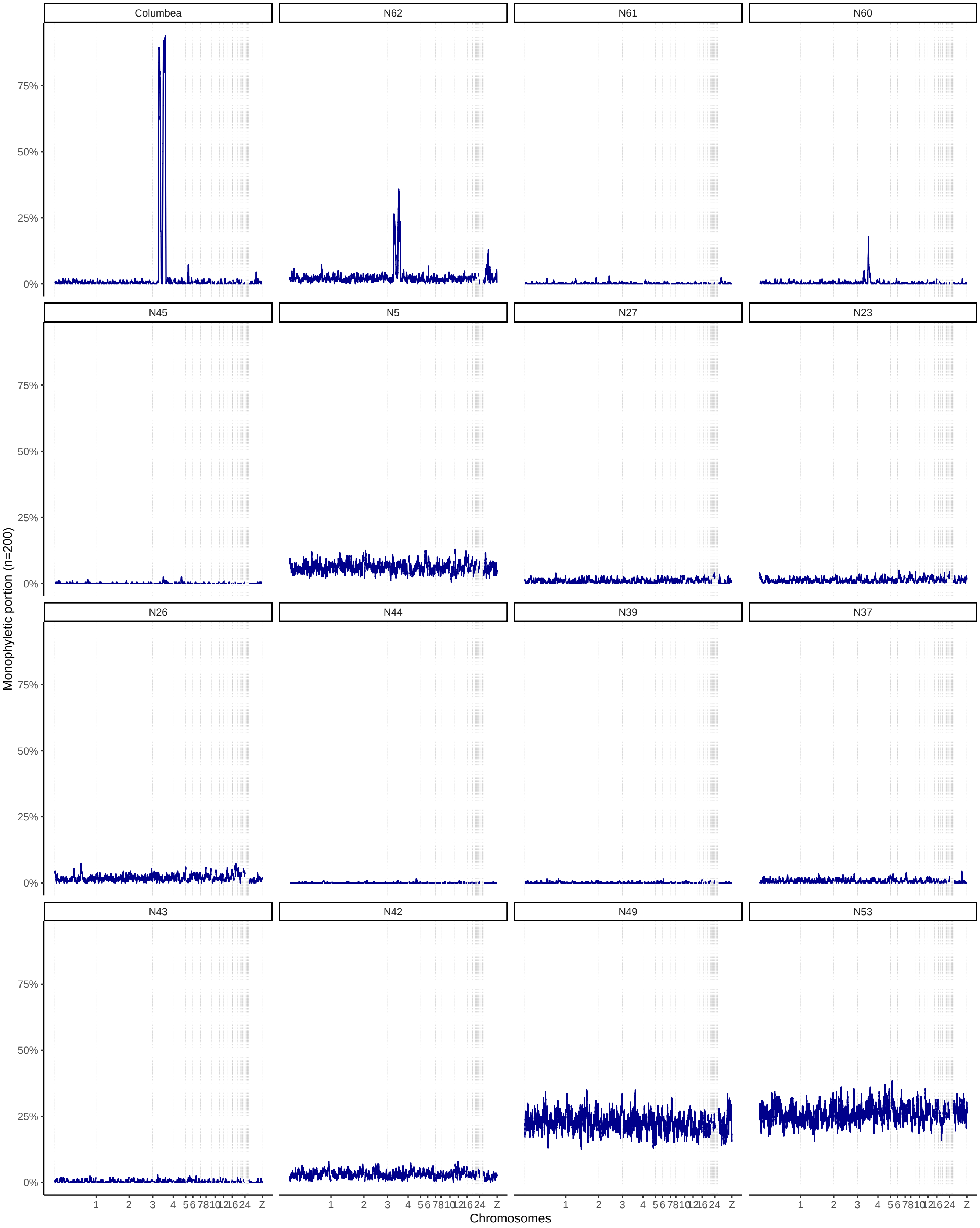

Fig. S6. The moving average of the recovery of the 16 focal clades as monophyletic (encoding 1 for monophyly, 0 for lack of monophyly) for 200 consecutive loci across different chromosomes for selected clades (panels), as labeled in Figure 1C (panels). Only loci are included that have at least one taxon from each of the two children of each node and one taxon from the sister group, and one taxon from outside the all these groups. The larger chromosomes are labeled at the bottom. Most high-ILS branches are rarely monophyletic, except for Columbea at the outlier region.

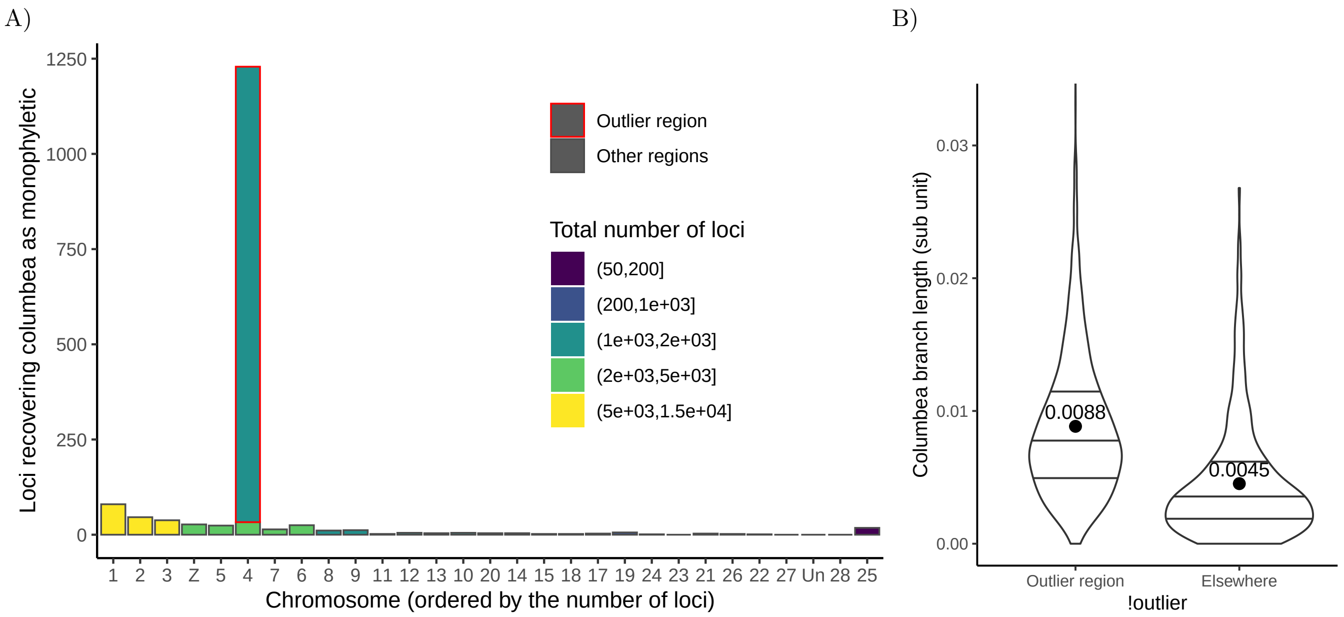

Fig. S7. A) The distribution of loci recovering Columbea as monophyletic across the genome. For each chromosome, the color indicates the number of loci in that chromosome. For chromosome 4, we separate the outlier region from the rest. B) The distribution of the branch uniting Columbea among locus trees that recover it as monophyletic and include at least one Columbimorphae, one Mirandornithes, and one Passera. The dot and the number show the mean (standard error bars are too small to be visible). $n = 372$ loci outside the outlier region and $n = 1197$ for loci in the outlier regions. The horizontal lines show the quartiles. The y-axis is cut at 0.035, leaving out five data points with branch lengths high than 0.035; these loci are not omitted form the the reported statistics.

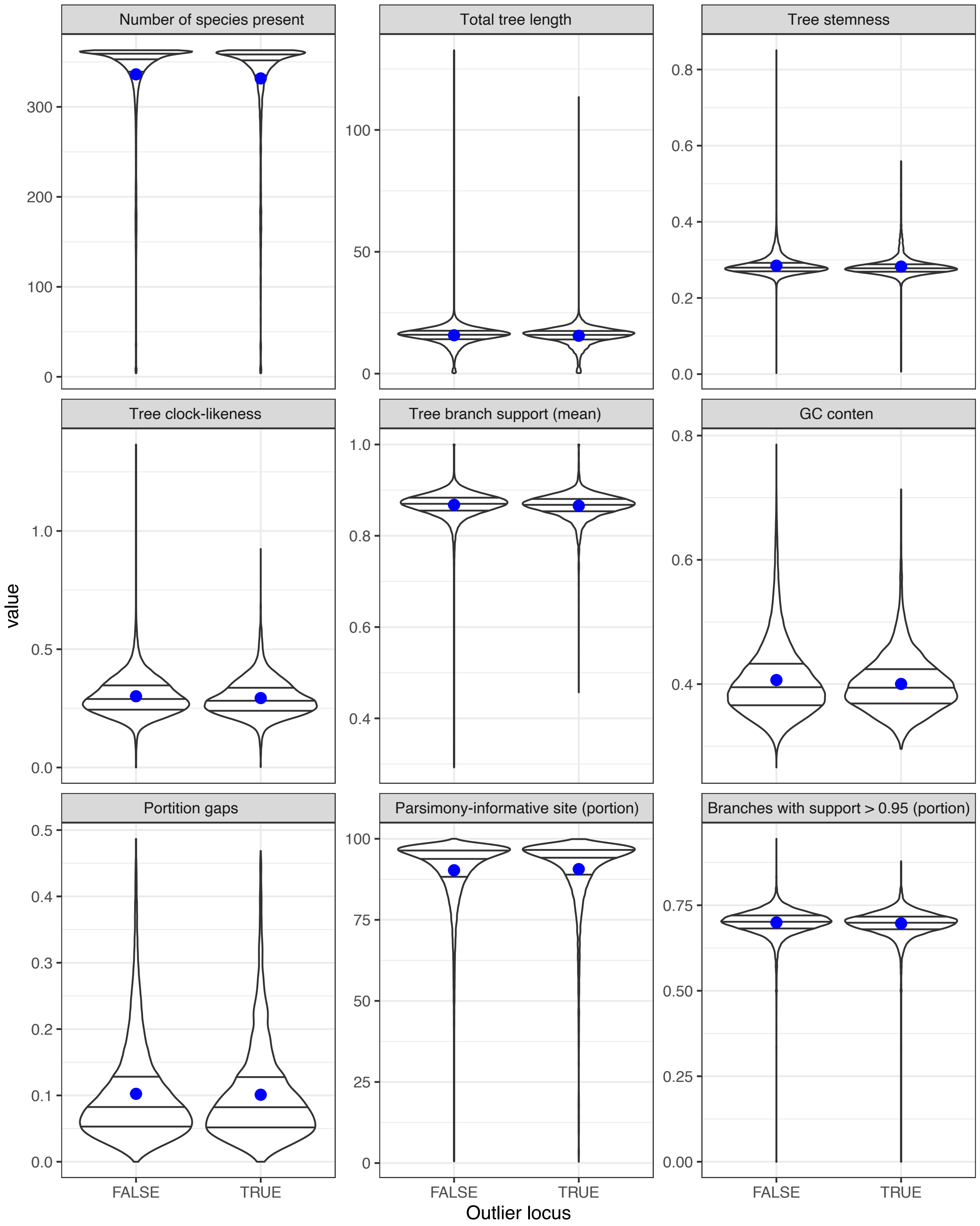

Fig. S8. Properties of locus trees that do or do not fall within the outlier region show no particular difference between the two groups. The number of species present, the length of the trees, tree stemness, clock-likeness, mean locus tree branch support , GC content, Portion of gaps, The portion of sites that are parsimony-informative, and the proportion of branches that have support above 0.95 show no clear distinction between loci in the outgroup region and other loci. Violin plots have the quantiles marked and blue dotes show mean and standard error (too small to be visible).

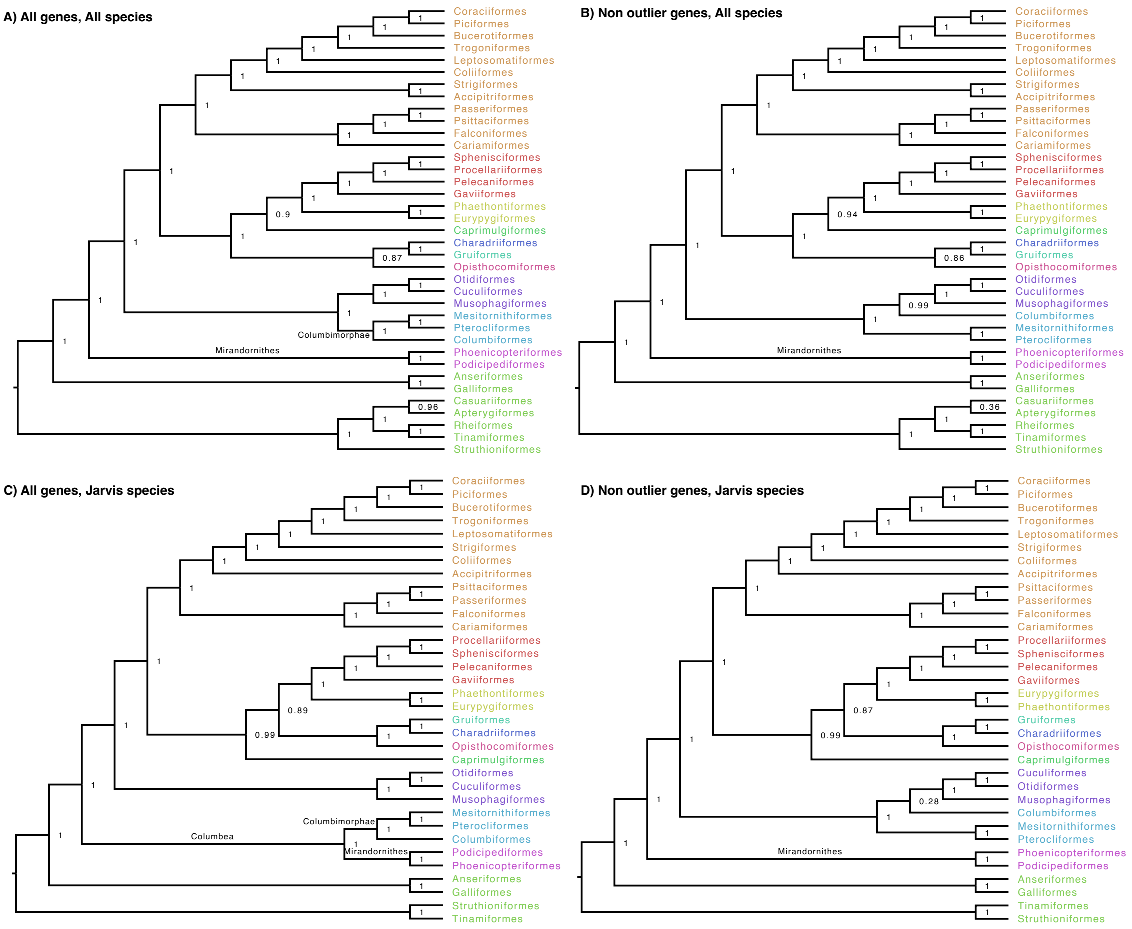

Fig. S9. Top) ASTRAL species trees inferred with (A,C) outlier locus trees or without (B,D) and with all species (A,B) or only restricted to the Jarvis 48 species (C,D).

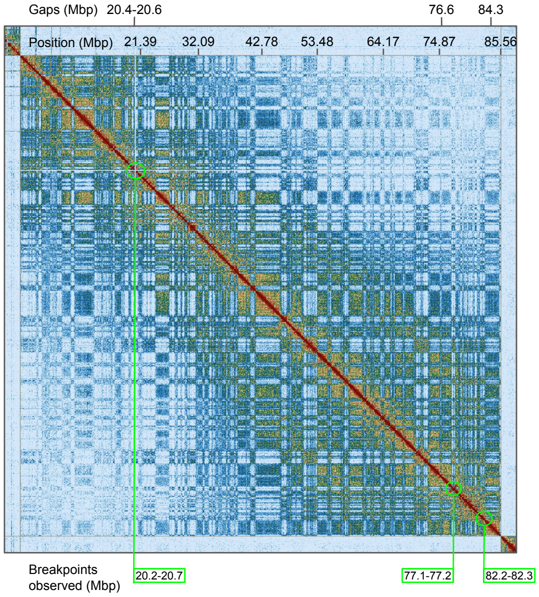

Fig. S10. The Hi-C interaction maps for the Turaco assembly. The reads are mapped using the Arima pipeline and the map is generated using PretextMap. Strong Hi-C interactions are indicated by red/orange/yellow coloring. We saw no clear indication of mis-assembly; if this was a mis-assembly, such that the region is inverted or at the wrong end of the chromosome, we would expect the interactions would look different. The gap track highlights gaps near the breakpoints of the turaco genome (bTauEry1). We observe a gap that corresponds to one of the breakpoints; however, there is strong interaction on either side of the gap suggesting contiguity, and thus the correct order. The other breakpoints do not contain gaps.

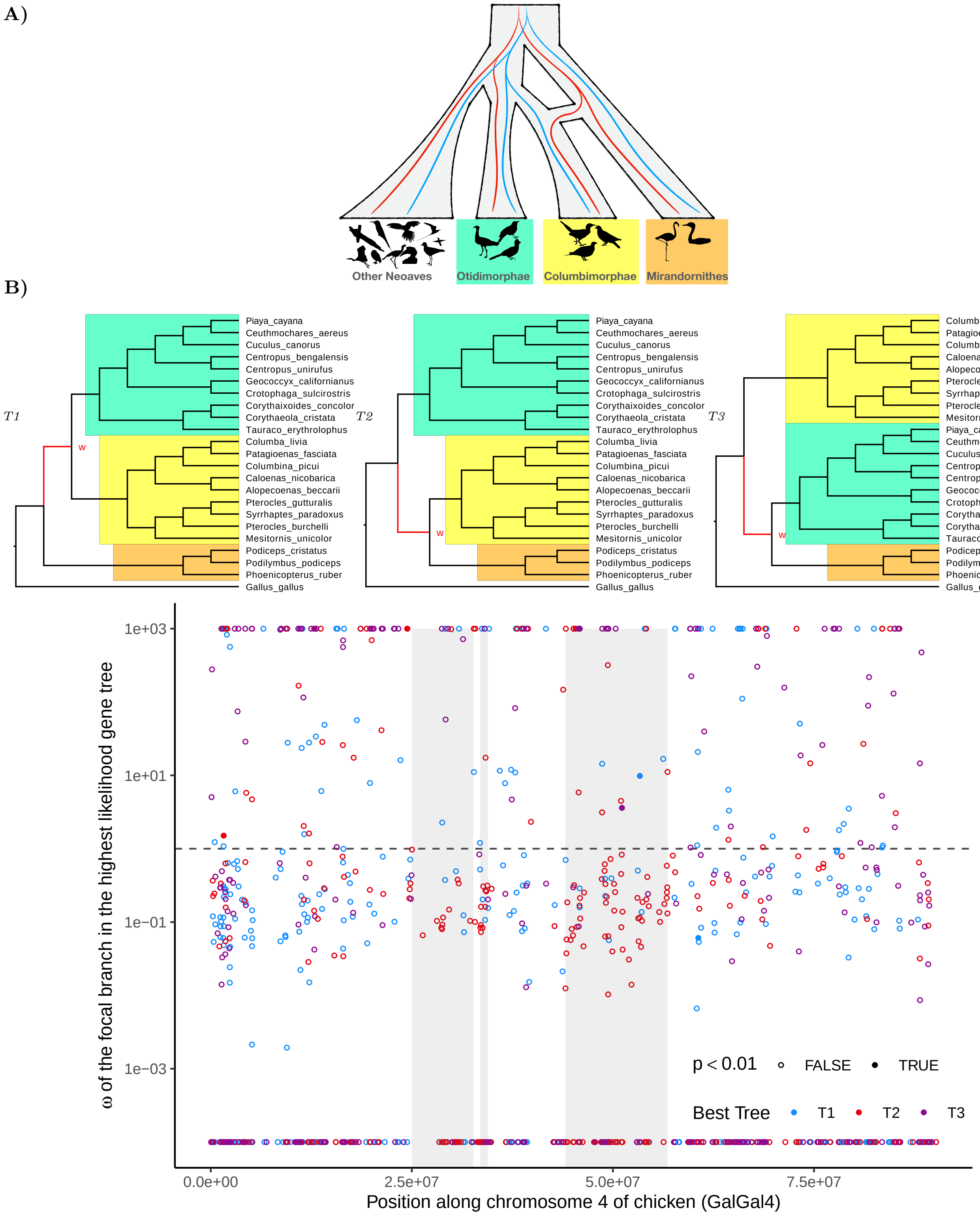

Fig. S11. A hybridization+selection explanation of the patterns. A) The hypothesis involves gene flow from the ancestor of Mirandornithes into the ancestor of Columbimorphae. The dominant evolutionary history is through the vertical edges, corresponding to lineage trees like the one in blue. However, for the outlier regions, the evolutionary history takes the lateral path, as shown in the red lineage tree. Note that the gene flow is directional from Mirandornithes to Columbimorphae and not vice versa (e.g., if a small number of individuals from Mirandornithes had constant reproductive contact with a much larger population of ancestral Columbimorphae). Since genes borrowed from Mirandornithes into Columbimorphae are concentrated in one region of chromosome 4, the gene flow could not have been random and must have been followed by a strong positive selection for borrowed genes. B) We found no sign of such strong positive selection in the outlier region. We computed the log-likelihood using PAML (1) for three locus tree topologies, as shown. We score all three trees under two scenarios: a single ω or a two-ω scenario with a background parameter and a "foreground" ω parameter on the focal branch, shown in red and marked with a w for each tree. For each gene along chromosome 4, we show the ω of the highest scoring of the three topologies, distinguishing the topology with colors. $\omega \gg 1$ indicates strong positive selection. We also show whether the p-value of the likelihood ratio test, comparing the two-ω model with the simpler single-ω model, is below 0.01. The outlier regions (shown in shades) do not stand out in terms of ω or cases with two-ω model being significantly better than the single-ω model.

 Mirarab *et al.*

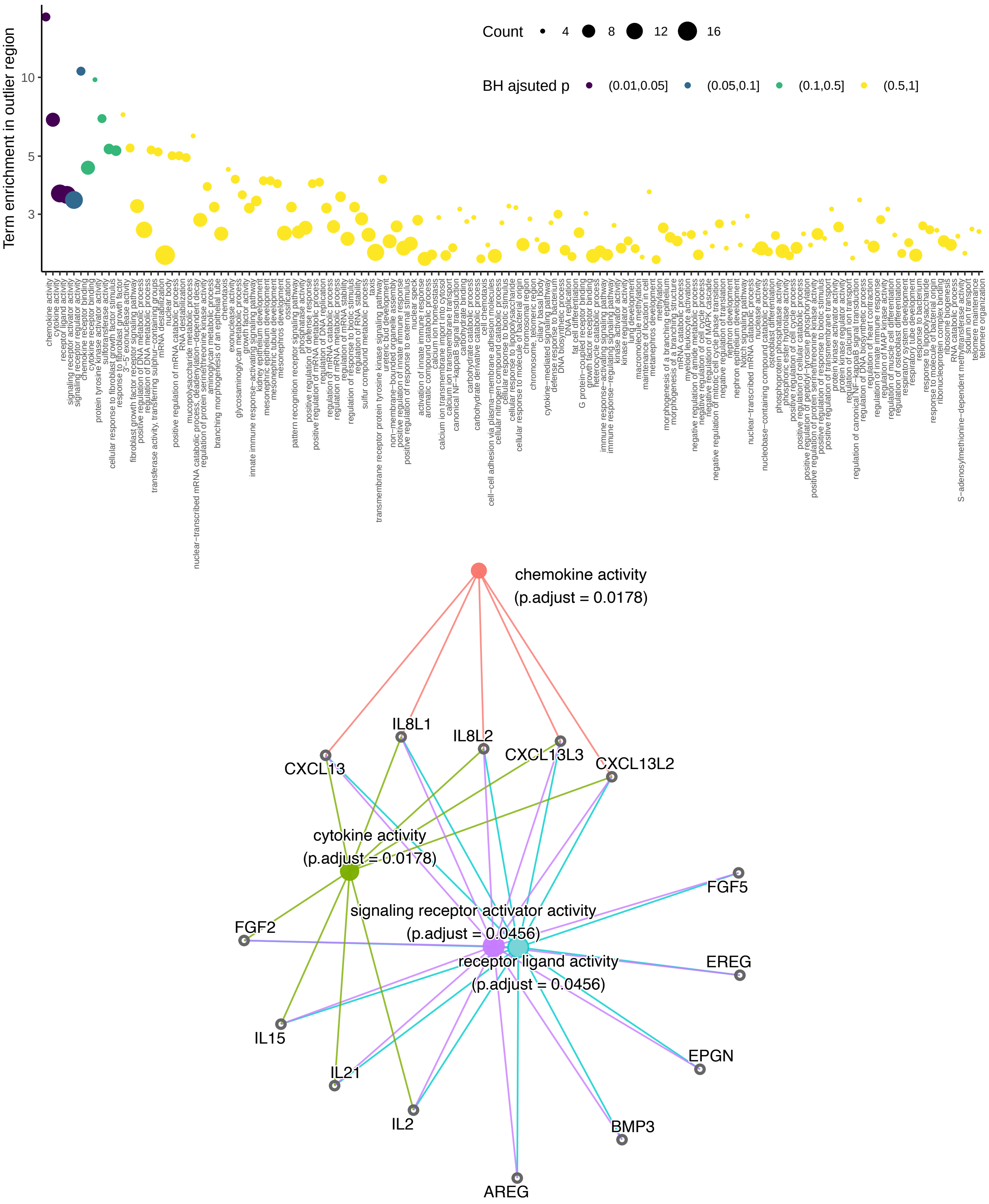

Fig. S12. GO Enrichment analysis of 352 genes located in the outlier region of chromosome 4. Top: Enrichment is defined as the relative frequency of the term in the outlier region divided by the relative frequency across the genome. For 134 GO terms that appear in the outlier region at least four times and are enriched by a factor of at least two, we show their enrichment (y-axis), their raw count (size), and the BH adjusted p-value for the enrichment test. Bottom: Four significantly enriched GO terms (BH-adjusted $p < 0.05$) in the molecular function (MF) category are shown, together with their associated genes and BH adjusted p values. The four terms are chemokine activity (GO:0008009), cytokine activity (GO:0005125), receptor ligand activity (GO:0048018), and signaling receptor activator activity (GO:0030546).

References

1. Z. Yang. PAML 4: Phylogenetic Analysis by Maximum Likelihood. *Molecular Biology and Evolution*, 24(8):1586–1591, April 2007. ISSN 0737-4038, 1537-1719. .
